# Cuproptosis Facilitates Chronic Skin Inflammation by Regulating the α‐Ketoglutarate/H3K4me3/Ferritin Heavy Chain 1 Signaling Pathway‐Mediated Ferroptosis

**DOI:** 10.1002/mco2.70425

**Published:** 2025-10-07

**Authors:** Pian Yu, Kaixuan Li, Shifu Luo, Rongxuan Yan, Xiaoqing Yi, Chi Fang, Sihui Ma, Guanming Wang, Fanyan Luo, Xiang Chen, Cong Peng, Jie Li

**Affiliations:** ^1^ The Department of Dermatology Xiangya Hospital Central South University Changsha China; ^2^ Hunan Key Laboratory of Skin Cancer and Psoriasis Hunan Engineering Research Center of Skin Health and Disease Xiangya Hospital Central South University Changsha China; ^3^ Furong Laboratory Changsha China; ^4^ National Engineering Research Center of Personalized Diagnostic and Therapeutic Technology Changsha China; ^5^ National Clinical Research Center for Geriatric Disorders Xiangya Hospital Central South University Changsha China; ^6^ The Department of Cardiovascular Surgery Xiangya Hospital Central South University Changsha China; ^7^ Faculty of Health Sciences University of Macau Taipa China; ^8^ Shenzhen Institute of Advanced Technology Chinese Academy of Sciences Shenzhen China; ^9^ Faculty of Computer Science and Control Engineering Shenzhen University of Advanced Technology Shenzhen China

**Keywords:** cuproptosis, ferroptosis, fth1, h3k4me3, inflammatory skin diseases, slc31a1

## Abstract

Dysregulated copper homeostasis is implicated in inflammatory skin diseases such as psoriasis and atopic dermatitis (AD), but the role of cuproptosis remains poorly defined. This study aimed to elucidate the role and mechanism of cuproptosis in inflammatory skin diseases. Transcriptome analysis of patient lesions revealed significant alterations in cuproptosis‐related genes correlating with disease‐specific pathological features. These cuproptosis‐related gene expression signatures demonstrated strong clinical relevance to therapeutic efficacy in both psoriasis and AD cohorts. Functional validation using disease models showed that pharmacologically inhibiting cuproptosis with the copper chelator tetrathiomolybdate (TTM), or genetically knocking down the copper importer SLC31A1, effectively alleviated chronic skin inflammation and hallmark pathological changes induced by imiquimod (IMQ) or calcipotriol (MC903). Mechanistically, we uncovered that SLC31A1‐mediated cuproptosis promotes intracellular α‐ketoglutarate (α‐KG) accumulation, driving activation of the lysine demethylase KDM5B. Activated KDM5B specifically demethylates H3K4me3 marks at the promoter of the ferroptosis regulator ferritin heavy chain 1 (FTH1), suppressing its transcription and consequently sensitizing keratinocytes to ferroptotic cell death, thereby amplifying inflammatory tissue damage. Our findings establish a fundamental pathogenic SLC31A1/KDM5B/FTH1 molecular axis linking dysregulated copper metabolism and cuproptosis to ferroptosis execution in psoriasis and AD, providing significant mechanistic insights and pinpointing promising therapeutic targets for these refractory skin disorders.

## Graphical abstract

Cuproptosis plays critical roles in psoriasis and atopic dermatitis (AD). SLC31A1‐mediated cuproptosis increases α‐ketoglutarate, activating KDM5B, which demethylates H3K4me3 domains in FTH1, lowering its transcription and promoting ferroptosis, thus advancing inflammatory skin diseases. Knockdown of SLC31A1 alleviates skin inflammation by reducing cuproptosis.

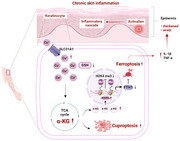

## 1 Introduction

Copper, classified as a trace metal element, is an essential element needed as a cofactor for the proper functioning of numerous enzymes in organisms. Copper plays a vital role in maintaining the overall functionality of cells in living organisms and is involved in numerous cellular processes that are closely related to cell fate, including oxidative phosphorylation, aerobic respiration, and cell growth and development [1, 2, 3, 4]. Although copper plays vital roles in essential cellular processes, it is important that copper levels are maintained at carefully regulated low levels to avoid the potential harmful effects of excessive copper accumulation [5]. Copper levels exceeding the normal range can have detrimental effects on cells, ultimately leading to cell death.

Copper‐induced cellular death, called cuproptosis, is a complicated process and is involved in various biochemical reactions. When copper forms a chemical bond with copper ion‐binding proteins, this copper–protein complex has the ability to generate reactive oxygen species (ROS), which contribute to the phenomenon of oxidative stress, causing potential harm to cellular components and biomolecules such as DNA [6]. Cuproptosis occurs through the direct combination of copper and the fatty acylation component of the tricarboxylic acid (TCA) cycle, which leads to the aggregation of fatty acylated proteins and the loss of iron sulfur (Fe/S) cluster proteins, triggering protein toxicity stress and ultimately leading to cell death [7].

Copper has been well documented to play crucial roles in skin function, particularly the intricate process of skin keratinization that is integral to maintaining skin health and integrity. Disruptions in copper homeostasis can lead to the onset of inflammatory skin diseases [8]. Previous studies have indicated that the serum copper level of psoriasis patients was significantly higher than that of healthy people [9, 10] and found a positive correlation between copper ion levels and the severity of psoriasis [11]. In addition, multiple studies have consistently demonstrated elevated copper levels in the hair of children with atopic dermatitis (AD), indicating a correlation between the disease and increased copper levels. Moreover, evidence has also shown a significant increase in serum ceruloplasmin levels among children diagnosed with AD [12]. However, it is still unclear whether cuproptosis contributes to the pathogenesis of skin diseases, particularly psoriasis and AD.

Psoriasis and AD are two chronic inflammatory skin diseases that share several similarities in their pathogenesis [13, 14, 15]. Both conditions involve dysregulation of the immune system, specifically the T‐cell‐mediated immune response. In psoriasis, the immune system mistakenly activates T cells, causing them to release inflammatory molecules such as cytokines. These chemicals trigger an inflammatory response in the skin, leading to the characteristic symptoms of the disease. Psoriasis is a chronic autoimmune condition characterized by erythema and scale on the skin. On the other hand, AD is another chronic inflammatory skin disorder that is often referred to as eczema, characterized by erythema and itchy on the skin. Significantly, psoriasis and AD share common features of dysfunction of keratinocytes (KCs) and the Th17‐mediated immune response [16, 17, 18, 19, 20].

In this study, we found that cuproptosis‐related genes, including SLC31A1 and ATP7A/B, were significantly altered in psoriasis and AD patients according to transcriptome data analysis. In addition, cuproptosis and the level of copper ions were increased in psoriasis and AD mouse model skin lesions. Moreover, inhibition or knockdown of SLC31A1 alleviated calcipotriol (MC903)‐induced AD‐like or imiquimod (IMQ)‐induced psoriasis‐like dermatitis in mice, indicating that copper homeostasis plays an important role in skin dermatitis. Interestingly, cuproptosis could significantly downregulate ferritin heavy chain 1 (FTH1) expression by promoting KDM5B‐mediated H3K4me3 demethylation, thereby inducing ferroptosis and ultimately promoting skin inflammation.

## 2 Results

### 2.1 Cuproptosis Is Involved in the Pathogenesis of Psoriasis and AD

We used published single‐cell RNA sequencing data (GSE147424 and GSE151177) to comprehensively analyze the single‐cell transcriptome data of psoriasis and AD patients (Figure S1A) [21, 22]. First‐level analysis showed 14 clusters (Figure S1B). Cell types included adipocytes, dendritic cells, fibroblasts, basale KCs, corneum KCs, granulosum and spinosum KCs, lymphatic endothelial cells, macrophages, melanocytes, PC‐cSMCs, sweat gland cells, T cells, and vascular endothelial cells (Figure S1C,D). In psoriasis, corneum KCs dominate, while basal KCs are the main cellular component in AD (Figure S1E).

**FIGURE 1:**
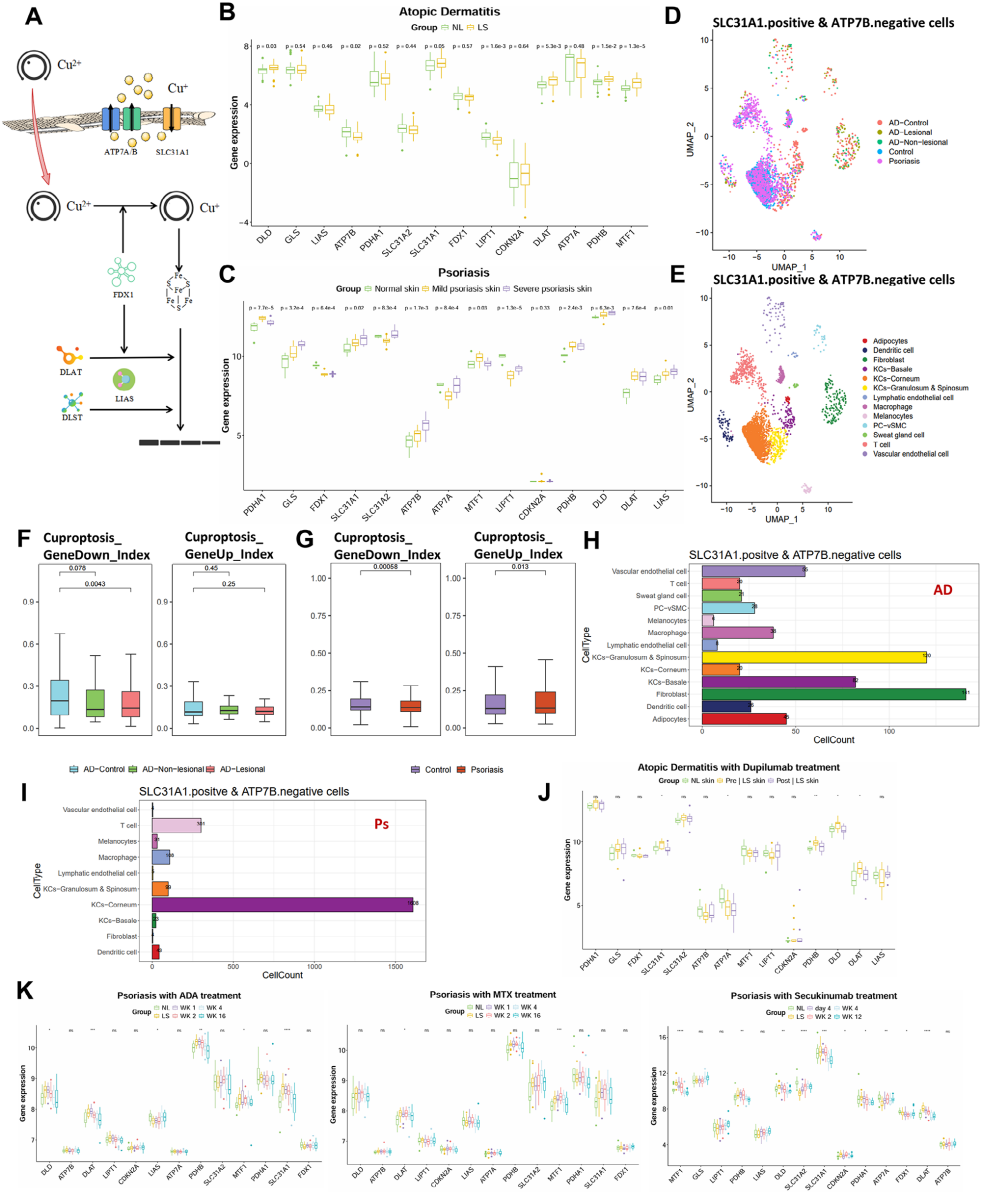
Analysis of cuproptosis‐related indicators. (A) Cuproptosis mechanism diagram. (B) The boxplot of cuproptosis‐related genes expressions in AD patients. (C) The boxplot of cuproptosis‐related genes expressions in psoriasis patients. (D) The UMAP plot of imbalanced copper homeostasis cells in AD and psoriasis (group by samples). (E) The UMAP plot of imbalanced copper homeostasis cells in AD and psoriasis (group by cell types). (F) The boxplot of cuproptosis‐GeneUp score and Cuproptosis‐GeneDown score of imbalanced copper homeostasis cells in AD. (G) The boxplot of cuproptosis‐GeneUp score and Cuproptosis‐GeneDown score of imbalanced copper homeostasis cells in psoriasis. (H) The number of different cell types exhibiting imbalanced copper homeostasis in AD. (I) The number of different cell types exhibiting imbalanced copper homeostasis in psoriasis. (J) The changes of cuproptosis‐related genes expressions in AD patients with Dupilumab treatment. (K) The changes of cuproptosis‐related genes expressions in psoriasis patients with different treatment. *p < 0.05, **p < 0.01, ***p < 0.001, ****p < 0.0001.

Excess copper directly binds to sulfhydryl proteins in the mitochondrial TCA cycle, causing abnormal aggregation of sulfhydryl proteins and loss of iron–sulfur cluster proteins in the respiratory chain complexes, triggering the protein toxicity stress response and ultimately leading to cell death [7] (Figure 1A). Analysis of transcriptome datasets (GSE277961 and GSE78097) from patients with psoriasis and AD revealed statistically significant differences in the expression of cuproptosis‐related genes (including SLC31A1, FDX1, LIPT1, and DLAT) in lesional skin tissues from PSO and AD patients compared to normal tissues, particularly in the elevation of SLC31A1 (Figure 1B,C). The copper transporter protein SLC31A1 plays a pivotal role in cuproptosis by facilitating the influx of copper ions into cells [23, 24]. In contrast, ATP7B, the copper transporter protein responsible for the efflux of copper ions, acts as the primary pathway for eliminating excess copper ions from the cell. The imbalanced expression of SLC31A1 (high) and ATP7B (low) in skin cells disrupts the normal equilibrium between copper influx and efflux. This disruption results in the accumulation of copper ions within the cell, subsequently leading to the initiation of cuproptosis. In scRNA‐seq analysis, we defined cells with imbalanced copper homeostasis as those displaying a relative expression level of SLC31A1 greater than 1 and a level of ATP7B lower than 1. Cells with imbalanced copper homeostasis were identified, and we proceeded to separate them from their respective cell clusters for further analysis and study (Figure 1D,E). FDX1, LIAS, LIPT1, DLD, DLAT, PDHA1, PDHB, MTG1, GLS and CDKN2A were defined as signature of cuproptosis. Specifically, given the abnormal aggregation of sulfhydryl proteins and loss of iron–sulfur cluster proteins in cuproptosis, FDX1, LIAS, LIPT1, DLD, DLAT, PDHA1, and PDHB were considered GeneDown genes, and GLS, CDKN2A, and MTF1 were considered GeneUp genes [7]. In AD patients, the score of the GeneDown genes in cells with imbalanced copper homeostasis of normal skin was higher than that in lesions (Figure 1F). Similarly, in psoriatic patients, the score of the GeneDown genes in cells with imbalanced copper homeostasis of normal skin was higher than that in lesions and the expression of the GeneUp genes in cells with imbalanced copper homeostasis of normal skin was lower than that in lesions (Figure 1G). These results above suggested the occurrence of cuproptosis in both AD and psoriasis. Subsequently, we conducted separate analyses of the AD and psoriasis data. In AD patients, fibroblasts exhibited a higher proportion of copper homeostasis imbalance, followed by granulosum and spinosum KCs (Figure 1H). In psoriasis, corneum KCs were overwhelmingly dominant, suggesting a significant role of KCs in copper homeostasis imbalance (Figure 1I). Immunofluorescence staining demonstrated significant expression of Slc31a1 localized to KCs. Furthermore, Slc31a1 expression levels were significantly upregulated in both PSO and AD disease model mice compared to normal controls (Figure S1F). Adalimumab (ADA), methotrexate (MTX), and secukinumab are classic drugs for treating psoriasis, while dupilumab is a commonly used drug for treating AD. Transcriptome data of lesional skin samples from treated patients (GSE85034, GSE137218, and GSE59294) revealed that SLC31A1 expression progressively decreased over the treatment period in psoriasis patients receiving ADA, MTX, or secukinumab. In AD patients treated with dupilumab, SLC31A1 expression declined from elevated levels to pre‐treatment baseline. This indicates that SLC31A1 expression levels may serve as a monitoring indicator for treatment response in both PSO and AD (Figure 1J–K).

### 2.2 Blocking Cuproptosis Alleviates Skin Inflammation

**FIGURE 2:**
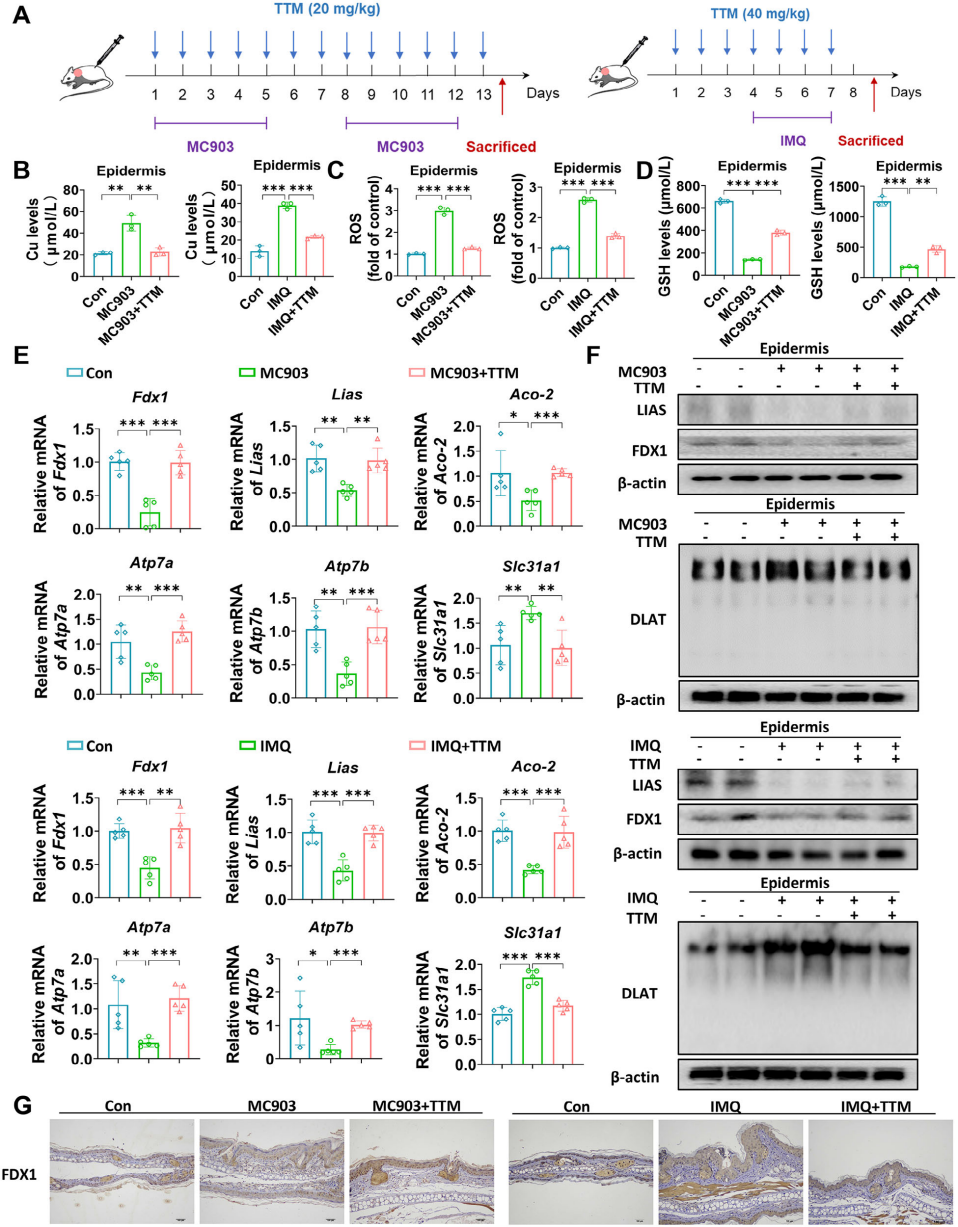
TTM alleviates cuproptosis in AD and psoriasis‐like dermatitis. (A) Mice were treated with ethanol and MC903 for 12 days (discontinued on days 6 and 7) to construct the AD‐like dermatitis model. Mice were treated with Vaseline and IMQ for 7 days to establish psoriasis‐like dermatitis. Mice were treated with TTM (20 mg/kg in AD, 40 mg/kg in psoriasis) by intragastric administration every day. (B) Copper ion levels in each group. (C) ROS levels in each group. (D) GSH levels in each group. (E) Transcription levels of iron–sulfur cluster proteins (Fdx1, Lias, and Aco‐2) and copper ion channels (Atp7a, Atp7b, and Slc31a1) in each group by qRT‐PCR. (F) Protein levels of iron–sulfur cluster proteins (FDX1 and LIAS) and the oligomerization of lipidated protein (DLAT) in each group by western blotting. (G) Expression of FDX1 in mice detected by IHC. *n* = 5. Scale bar = 200 µm. Graphs indicate the mean ± SD of each group. *p<0.05, **p<0.01, ***p<0.001.

To further investigate the effect of cuproptosis on dermatitis, we administered a copper ion chelator tetrathiomolybdate (TTM) in a mouse skin inflammatory model (Figure 2A). Our findings indicated that TTM treatment effectively decreased the concentrations of copper and ROS in the skin lesion tissue of MC903‐ and IMQ‐induced dermatitis, whereas significantly elevated the levels of glutathione (GSH) (Figure 2B,D). TTM treatment has been shown to rescue the reduction of iron–sulfur cluster proteins, such as Fdx1, Lias, and Aco‐2, and TTM could increase the expression Atp7a/b and reduce the expression of Slc31a1 mRNA after treated with MC903 and IMQ (Figure 2E). Additionally, TTM treatment led to a notable increase in the expression levels of the iron–sulfur cluster proteins LIAS and FDX1, while the oligomerization of the sulfur‐transfer protein DLAT was decreased (Figure 2F). Furthermore, our study highlighted the critical role of Fdx1 in cuproptosis. Moreover, we further confirmed that FDX1 expression was downregulated in KCs from both AD and psoriasis disease models. Following TTM intervention, its expression levels were significantly restored (Figure 2G; Figure S2A).

**FIGURE 3:**
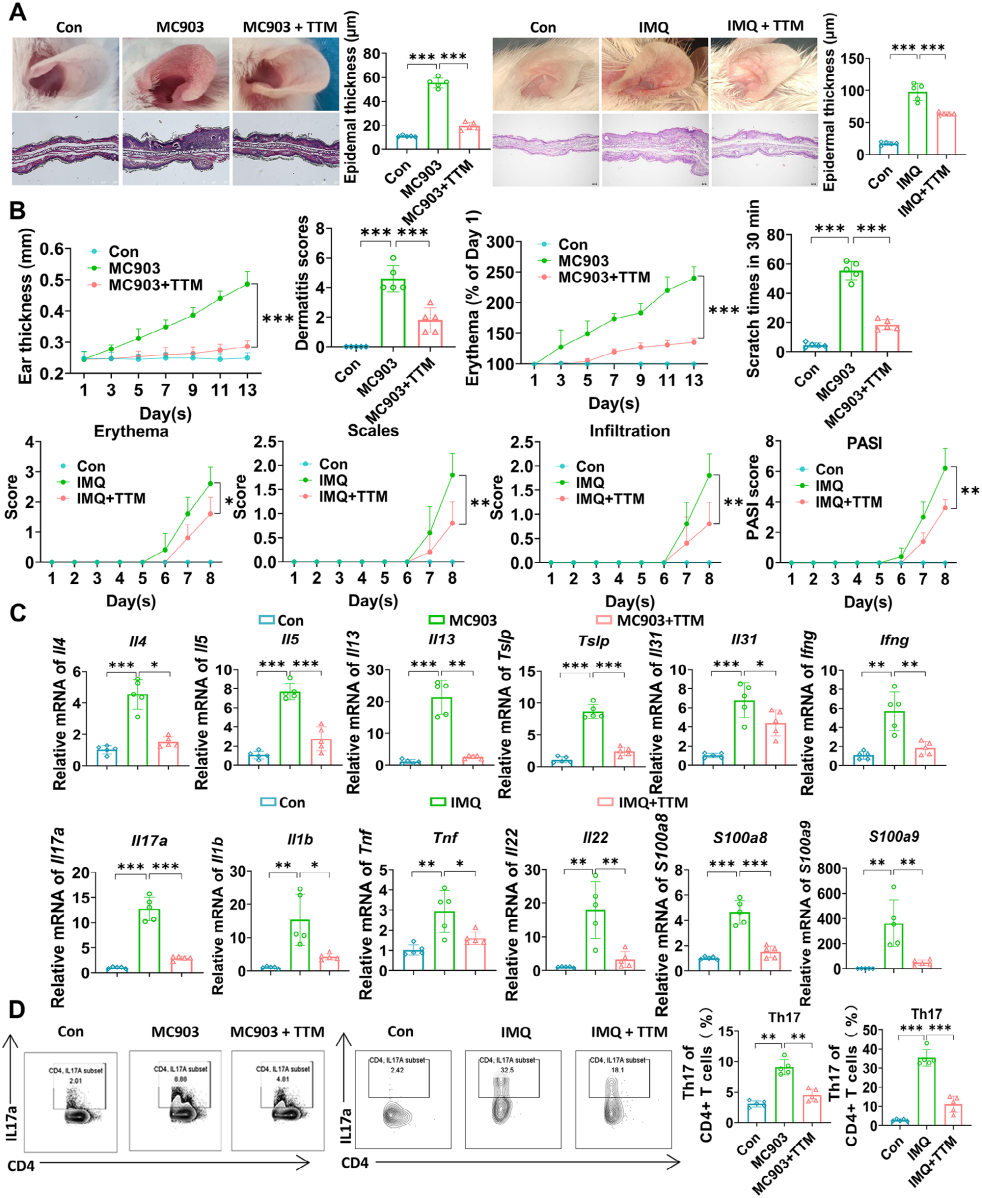
TTM alleviate AD and psoriasis‐like dermatitis. (A) Mice were treated with ethanol and MC903 for 12 days (discontinued on days 6 and 7) to construct the AD‐like dermatitis model. Mice were treated with Vaseline and IMQ for 7 days to establish psoriasis‐like dermatitis. Mice were treated with TTM (20 mg/kg in AD, 40 mg/kg in psoriasis) by intragastric administration every day. Phenotypic presentation and H&E staining as well as statistical analysis of the epidermal thickness of the ears in mice. Scale bars = 200 µm. (B) The severity scoring of skin lesions, the ear thickness, dermatitis scores, erythema, and scratch times in 30 min as well as the statistical analysis were used to evaluate AD. The erythema, scales, infiltration, and PASI scores were used for evaluating psoriasis. (C) mRNA levels of inflammatory factors (Il4, Il5, Il13, Tslp, Il31, and Ifng were used for evaluating AD; Il17a, Il1b, Tnf, Il22, S100a8, and S100a9 were used for evaluating psoriasis) associated with dermatitis in each group by qRT‐PCR. (D) Representative flow cytometric plots and quantification of the Th17 cell percentage gated on the CD4^+^ T cells in each group. *n* = 5. *p < 0.05, **p < 0.01, ***p < 0.001.

Remarkably, the cuproptosis inhibitor TTM suppressed MC903‐induced dermatitis, including epidermal thickness, ear thickness, dermatitis score, erythema, and the number of scratches (Figure 3A,B). Similarly, in IMQ‐induced skin inflammatory responses, TTM exhibited notable effects in reducing erythema, scale, infiltration, and PASI scores (Figure 3A,B). Furthermore, TTM suppressed the transcription levels of inflammatory and chemotactic factors in the mouse epidermis, such as AD‐related genes Il4, Il5, Il13, Tslp, Il31, and Ifng, and psoriasis‐related genes Il7a, Il1b, Tnf, Il22, S100a8, and S100a9 (Figure 3C). In addition, TTM also reduced the spleen index in mice with psoriasis and AD (Figure S2B). Additionally, TTM treatment remarkably suppressed the infiltration of Th17 cells (Figure 3D), which play a crucial role in the pathogenesis of these inflammatory skin conditions, suggesting that inhibition of cuproptosis may have potential anti‐inflammatory response properties. In addition, we found that TTM treatment did not have a significant impact on the body weight of the mice, indicating its favorable safety profile (Figure S2C). Histological analysis of sample tissues from the heart, liver, spleen, lung, and kidney revealed that TTM did not exert significant toxic effects on healthy tissues (Figure S2D).

### 2.3 SLC31A1‐Mediated Downregulation of FTH1 Is Involved in the Association Between Cuproptosis and Ferroptosis

**FIGURE 4:**
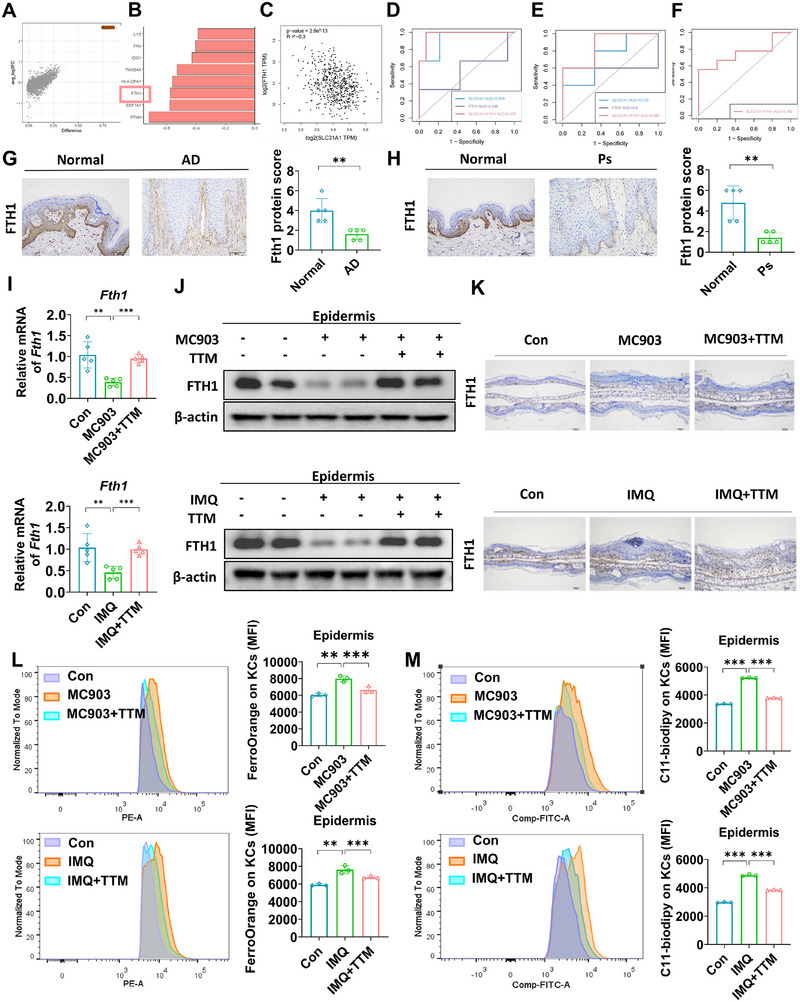
Fth1 is a bridge molecule for cross‐talk between cuproptosis and ferroptosis. (A) In KCs‐Corneum cells of psoriasis, the differentially expressed genes between imbalanced copper homeostasis cells and other cells. (B) The top eight differentially down‐expressed genes of (A). (C) The correlation analysis between FTH1 and SLC31A1 expression of normal skin tissues in GEPIA database. (D and E) The bivariate factor (SLC31A1 and FTH1) model evaluate by ROC curve in AD and psoriasis datasets. (F) The ROC curve of external validation of (E) in psoriasis dataset. (G) Expression of FTH1 in skin tissues of AD patient groups detected by IHC, scale bars = 200 µm, *n* = 5. (H) Expression of FTH1 in skin tissues of psoriasis patient groups detected by IHC, scale bars = 200 µm, *n* = 5. (I) Mice were treated with ethanol and MC903 for 12 days (discontinued on days 6 and 7) to construct the AD‐like dermatitis model. Mice were treated with Vaseline and IMQ for 7 days to establish psoriasis‐like dermatitis. Mice were treated with TTM (20 mg/kg in AD, 40 mg/kg in psoriasis) by intragastric administration every day. The mRNA level of Fth1 by qRT‐PCR in each group. (J) The protein level of FTH1 by western blotting in each group. (K) The expression of FTH1 detected by IHC in each group in mice, scale bars = 200 µm, *n* = 5; *p < 0.05, **p < 0.01, ***p < 0.001. (L) Fe^2+^ level detected by Ferrorange using flow cytometry in each group. (M) Lipid peroxidation levels by C11‐BODIPY581/591 using flow cytometry in each group. *n* = 3. *p < 0.05, **p < 0.01, ***p < 0.001.

We conducted a comprehensive analysis of scRNA data from psoriasis patients that compared cells displaying cuproptosis‐positive characteristics (SLC31A1> 1 and ATP7B < 1) with cells exhibiting cuproptosis‐negative features (SLC31A1< 1 and ATP7B > 1), specifically KCs. The results showed low expression of FTH1, a crucial inhibitory molecule for ferroptosis, in cuproptosis‐negative cells (Figure 4A,B), and FTH1 was also demonstrated to be negatively related to SLC31A1 expression (Figure 4C).

Next, we collected data from datasets related to AD and psoriasis treatments, and the results showed that the bivariate factor model incorporating SLC31A1 and FTH1 had superior predictive performance in determining the treatment effect of AD (GSE69967) (Figure 4D) [25]. Similarly, the application of the SLC31A1 + FTH1 bivariate model also showed an effective predictive indicator for psoriasis therapy outcome (GSE130588) (Figure 4E) [26]. To validate the model, we further evaluated its suitability using the treatment dataset of ADA (GSE85034) and verified its effectiveness as a prediction model (Figure 4F) [27]. Moreover, we observed a significant downregulation of FTH1 expression in AD and psoriasis patient skin lesions (Figure 4G,H). Figure S5A shows that si‐Slc31a1 could upregulate the Fth1 mRNA level in JB6 cells. In addition, in the dermatitis mouse model, TTM treatment reversed MC903‐ and IMQ‐induced FTH1 mRNA and protein downregulation (Figure 4I–K). Subsequently, we assessed the levels of Fe^2+^ and lipid peroxidation in epidermal cells and found that MC903 and IMQ treatment led to a significant increase in Fe^2+^ as well as lipid peroxidation in the mouse epidermis, which can be effectively reversed by TTM treatment (Figure 4L–M). Given that downregulation of FTH1 induces ferroptosis [28], and considering the well‐established consensus that ferroptosis drives the progression of inflammatory diseases [29], we extended our investigation to evaluate the interventional efficacy of ferroptosis inhibitor ferrostatin‐1 (Fer‐1) in AD (Figure S3A), building upon prior research demonstrating Fer‐1's inhibitory effect on psoriasis progression [30]. The results demonstrated that Fer‐1 significantly alleviated AD‐like phenotypes in disease models without affecting body weight (Figure S3B–D). Furthermore, Fer‐1 effectively suppressed the expression of AD‐associated inflammatory cytokines (Figure S3E). Thus, we provide evidence here that cuproptosis promotes psoriasis and AD progression by inducing FTH1‐mediated ferroptosis.

### 2.4 α‐Ketoglutarate (α‐KG) Induced Demethylation of H3K4me3 Leads to a Decrease in FTH1 Expression

**FIGURE 5:**
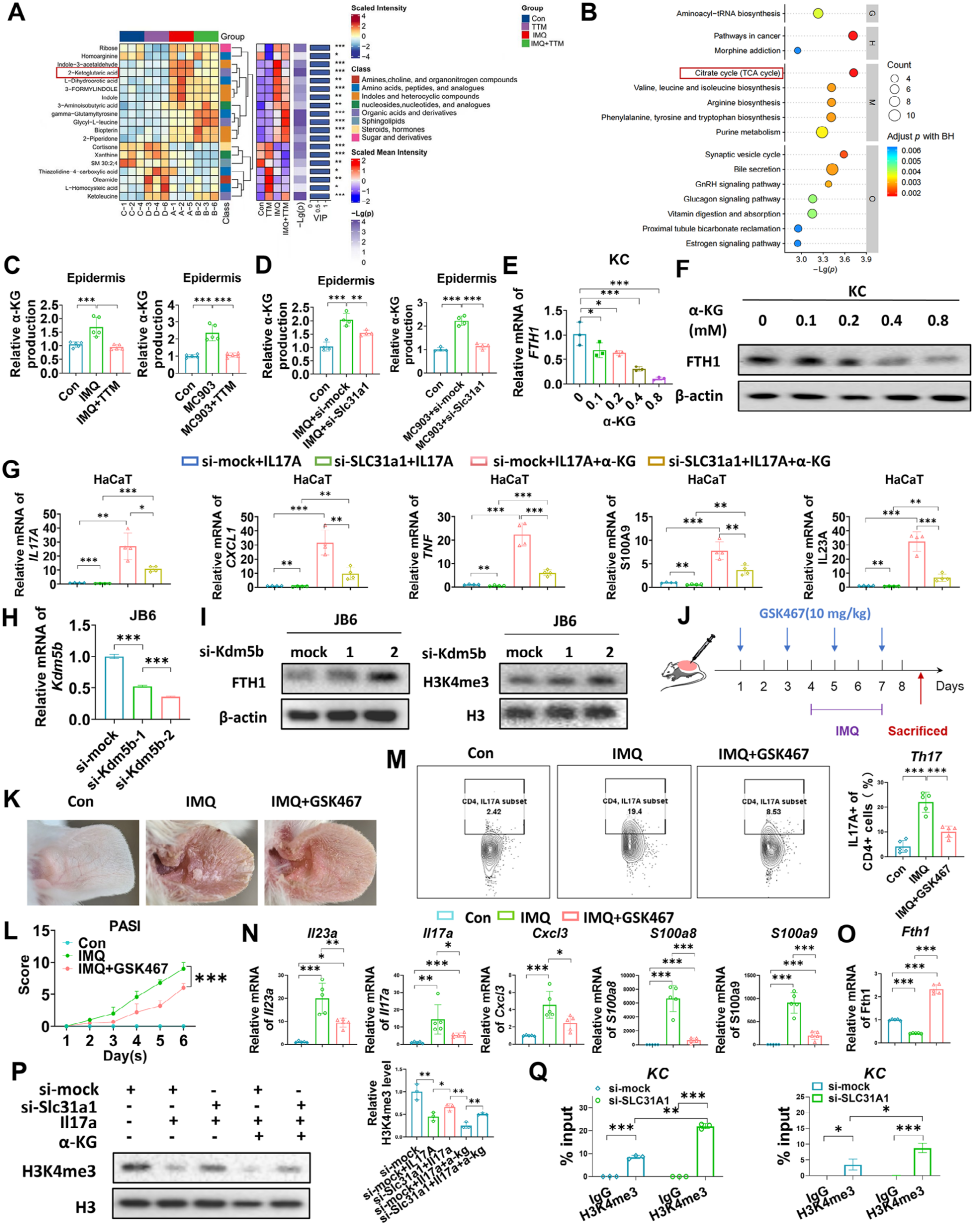
α‐KG inhibits FTH1 expression through demethylation of H3K4me3. (A) Mice were treated with ethanol and MC903 for 12 days (discontinued on days 6 and 7) to construct the AD‐like dermatitis model. Mice were treated with Vaseline and IMQ for 7 days to establish psoriasis‐like dermatitis. Metabolomics sequencing was performed on the ear tissue of mice after modeling. The complex heatmap of differential metabolites. (B) The KEGG clustering of differential metabolites. (C and D) Mice were treated with ethanol and MC903 for 12 days (discontinued on days 6 and 7) to construct the AD‐like dermatitis model. Mice were treated with Vaseline and IMQ for 7 days to establish psoriasis‐like dermatitis. Mice were treated with TTM (20 mg/kg in AD, 40 mg/kg in psoriasis) by intragastric administration every day. Si‐Slc31a1 was applied to the back skin (2.5 nmol) every other day. The content of epidermic α‐KG. (E and F) Primary KCs were extracted from human foreskin tissue, and KCs were treated with 0–0.8 mM α‐KG for 48 h; the expression of FTH1 was detected by qRT‐PCR and WB. (G) Inflammatory and chemotactic factors were detected by qRT‐PCR after HaCaT treating with si‐mock, si‐Slc31a1, IL17A, or α‐KG for 48 h. (H) JB6 cells were transfected with either the non‐targeting siRNA or Kdm5b siRNA. Kdm5b mRNA level was measured by qRT‐PCR. (I) JB6 cells were transfected with either the non‐targeting siRNA or Kdm5b siRNA. FTH1 protein level was measured by western blotting, β‐actin served as a loading control. H3K4me3 protein level was measured by western blotting, and H3 served as a loading control. (J) Mice were treated with Vaseline and IMQ for 7 days to establish psoriasis‐like dermatitis. GSK467 was injected into the abdomen every other day (10 mg/kg). Collected the epidermis tissue. (K) Mice were treated with Vaseline and IMQ for 7 days to establish psoriasis‐like dermatitis. GSK467 was injected into the abdomen every other day (10 mg/kg). Phenotypic presentation in mice. Scale bars = 200 µm; (L) The PASI scores were used to evaluate psoriasis. (M) Representative flow cytometric plots and quantification of the Th17 cell percentage gated on the CD4^+^ T cells in each group. *n* = 5. (N) mRNA levels of Il23a, Il17a, Cxcl3, S100a8, and S100a9. (O) mRNA level of Fth1. (P) The expression of H3K4me3 in KCs detected by western blotting after treating with si‐SLC31A1, IL17A, and α‐KG for 48 h. (Q) ChIP–qPCR assays for H3K4me3 binding to the FTH1 promoter sites in KCs with or without si‐SLC31A1 treatment; *n* = 3. *p < 0.05, **p < 0.01, ***p < 0.001.

Next, we performed metabolomic sequencing on the ear tissues of C57BL/6 mice with control, TTM, IMQ, and IMQ plus TTM. Qualitative and significant differences in metabolite expression levels were used to perform hierarchical clustering of the samples, allowing accurate screening of marker metabolites. The heat map shows that α‐KG is more prominent in differential metabolites, and the TCA cycle ranks among the top in the KEGG pathway enrichment bubble map. Therefore, it is speculated that α‐KG is a key factor in the regulation of FTH1 by cuproptosis in skin tissue (Figure 5A,B; Figure S4A–D). We further examined α‐KG content in mouse ears and found that α‐KG content was increased in mouse AD and psoriasis models compared with the control group, and TTM/si‐Slc31a1 reversed this effect (Figure 5C,D). In addition, KCs were extracted from human foreskin tissue and treated with si‐SLC31A1 and IL17A, and we found that α‐KG increased after adding IL17A, while α‐KG content decreased after knocking down SLC31A1 (Figure S4E). We further inflamed the regulatory effect of α‐KG on FTH1 in vitro. KCs were treated with α‐KG at an increasing concentration of 0.1–0.8 mM. The results showed that the protein and mRNA levels of FTH1 decreased with the increase of α‐KG concentration (Figure 5E–F). Similarly, we found that SLC31A1 knockdown resulted in a decrease in IL17A, CXCL1, TNF, S100A9, and IL23A after treating with IL17A. Co‐treatment of HaCaT with IL17A and α‐KG resulted in a significant increase in inflammatory and chemotactic factors, which could also be reduced by si‐Slc31a1 (Figure 5G). It has been reported that α‐KG‐dependent KDM5B plays a pivotal role in regulating levels of H3K4me3 histone modifications [31], so we hypothesized that SLC31A1‐mediated FTH1 suppression may be achieved by regulating α‐KG/KDM5B/H3K4me3 signaling pathway. We intervened JB6 cells with siRNA of Kdm5b, and Figure 5H shows that Kdm5b was significantly knocked down. In addition, knockdown of Kdm5b significantly upregulated the protein levels of H3K4me3 and Fth1 (Figure 5I). Meanwhile, the Kdm5b‐specific inhibitor GSK467 alleviated IMQ‐induced psoriatic dermatitis (Figure 5J–K), including erythema, scales, thickness, and PASI scores (Figure 5L; Figure S4F). Furthermore, GSK467 treatment had no significant effect on body weight in mice (Figure S4G). In addition, inhibition of kdm5b expression also inhibited inflammation of IMQ‐induced psoriasis mice, including Th17 cells infiltration (Figure 5M), and the expression of inflammatory factors (Il23a, Il17a, Cxcl3, S100a8, and S100a9) (Figure 5N). As expected, inhibition of Kdm5b increased mRNA expression of Fth1 (Figure 5O). To further verify the relationship between H3K4me3 and α‐KG in inflammatory skin diseases, KCs were treated with si‐SLC31A1, IL17A, and α‐KG. The results showed that IL17A stimulation and α‐KG treatment reduced the protein level of H3K4me3 in KCs, and si‐SLC31A1 inhibited this process (Figure 5P). Next, we analyzed the expression profile of H3K4me3 in the Fth1 promoter region using a public database, and then designed primers for chromatin immunoprecipitation (ChIP) assay (Figure S4H). Figure 5Q shows that H3K4me3 could directly bind to the promoter of FTH1, and si‐SLC31A1 treatment promoted their interaction in KCs, which was consistent with our speculation. Taken together, our results revealed that α‐KG can influence the demethylation of H3K4me3 through KDM5B, ultimately suppressing the transcription of FTH1.

### 2.5 Knockdown of SLC31A1 Attenuates Skin Inflammation Through Inhibition of Cuproptosis

**FIGURE 6:**
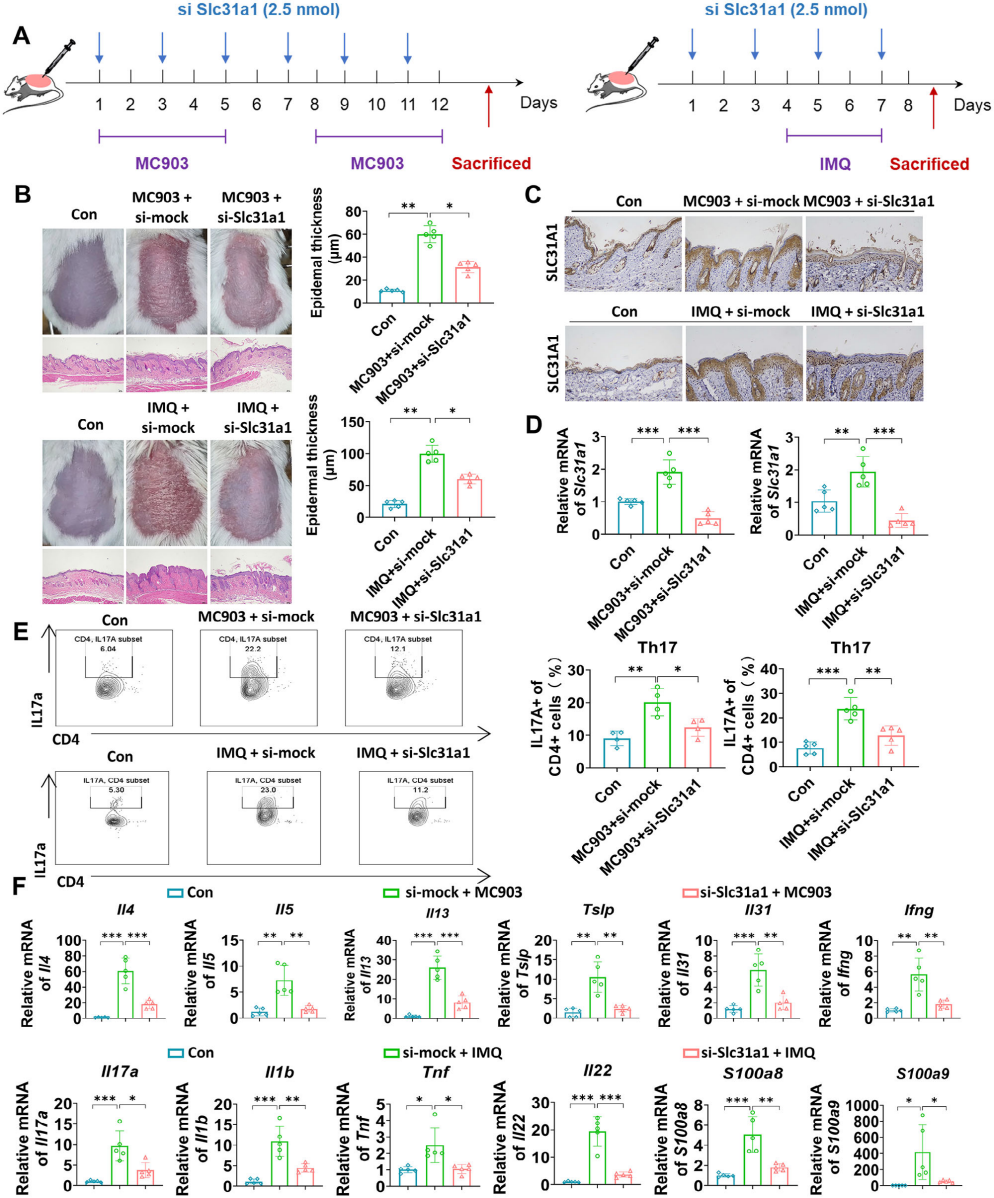
Knockdown of Slc31a1 alleviate AD and psoriasis‐like dermatitis. (A) Mice were treated with ethanol and MC903 for 12 days (discontinued on days 6 and 7) to construct the AD‐like dermatitis model. Mice were treated with Vaseline and IMQ for 7 days to establish psoriasis‐like dermatitis. Si‐Slc31a1 was applied to the back skin (2.5 nmol) every other day. (B) Phenotypic presentation and H&E staining as well as statistical analysis of the epidermal thickness of the back in mice. Scale bars = 200 µm, *n* = 5. (C) The expression of SLC31A1 in each group detected by IHC. Scale bars = 200 µm, *n* = 5. (D) The expression of epidermic Slc31a1 detected by qRT‐PCR in each group. (E) Representative flow cytometric plots and quantification of the Th17 percentage gated on the CD4^+^ T cells in each group. *n* = 5. (F) mRNA levels of inflammatory factors (Il4, Il5, Il13, Tslp, Il31, and Ifng were used for evaluating AD; Il17a, Il1b, Tnf, Il22, S100a8, and S100a9 were used for evaluating psoriasis) associated with dermatitis in each group by qRT‐PCR; *n* = 5. *p < 0.05, **p < 0.01, ***p < 0.001.

**FIGURE 7:**
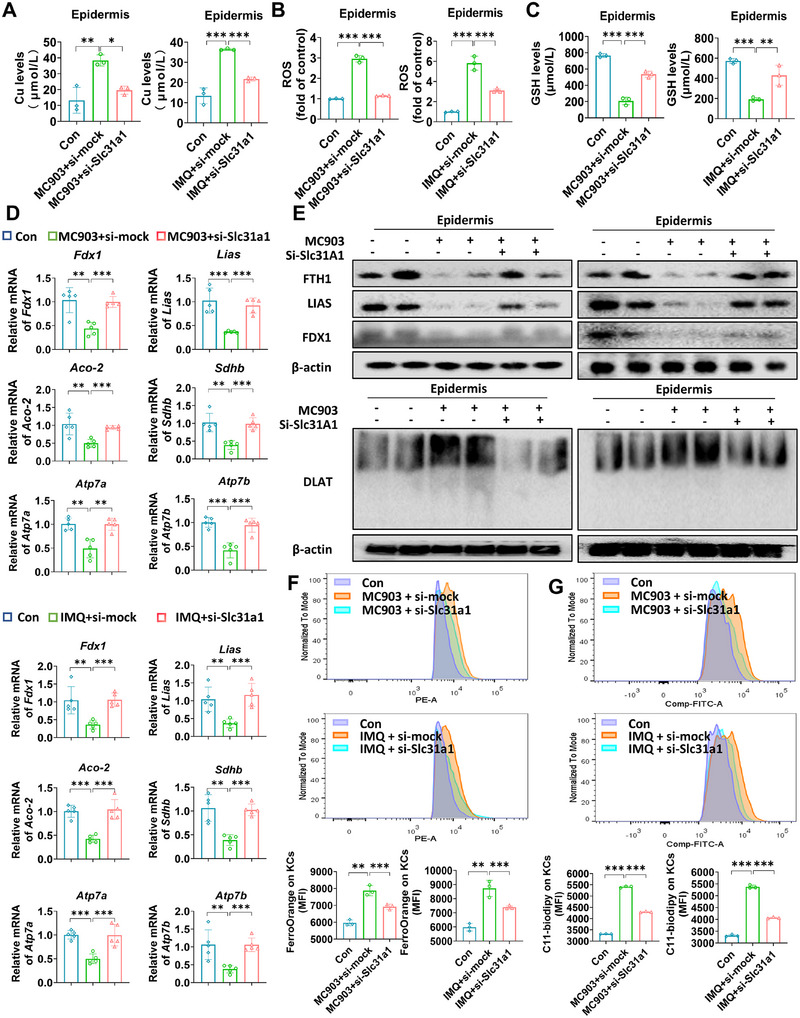
Knockdown of Slc31a1 could reduce cuproptosis in inflammatory skin diseases and inhibit ferroptosis through Fth1. (A) Mice were treated with ethanol and MC903 for 12 days (discontinued on days 6 and 7) to construct the AD‐like dermatitis model. Mice were treated with Vaseline and IMQ for 7 days to establish psoriasis‐like dermatitis. Si‐Slc31a1 was applied to the back skin (2.5 nmol). Copper ion levels in each group. (B) ROS levels in each group. (C) GSH levels in each group. (D) Transcription levels of iron–sulfur cluster proteins (Fdx1, Lias, Aco‐2, and Sdhb) and copper ion channels (Atp7a and Atp7b) in each group by qRT‐PCR. (E) Protein levels of iron–sulfur cluster proteins (FDX1 and LIAS), FTH1, and the oligomerization of lipidated protein (DLAT) in each group by western blotting. (F) Fe^2+^ level detected by Ferrorange using flow cytometry in each group. (G) Lipid peroxidation levels by C11‐BODIPY581/591 using flow cytometry in each group. *n* = 5. *p < 0.05, **p < 0.01, ***p < 0.001.

Given that cuproptosis may exert a critical role in skin inflammation and that SLC31A1 is highly expressed in skin lesions, we investigated the role of this molecule in chronic skin inflammation. siRNA targeting Slc31a1 was administered to the back skin of mice (Figure 6A), which suppressed MC903‐ and IMQ‐induced dermatitis and reduced epidermal thickness (Figure 6B). The expression of Slc31a1 was further validated by IHC (Figure 6C) and qRT‒PCR (Figure 6D). MC903 and IMQ treatment increased Slc31a1 expression, but it was substantially decreased after the administration of si‐Slc31a1. Furthermore, inhibition of Slc31a1 expression exhibited similar effects to TTM treatment, including blocking the infiltration of Th17 cells (Figure 6E), reducing the spleen index (Figure S5B), lowering the severity rating of dermatitis (Figure S5C), and suppressing the expression of inflammatory factors, such as AD‐related genes Il4, Il5, Il13, Tslp, Il31, and Ifng, and psoriasis‐related genes Il7a, Il1b, Tnf, Il22, S100a8, and S100a9 (Figure 6F). As expected, the suppression of Slc31a1 significantly reduced the elevated copper accumulation and the levels of ROS induced by MC903 (Figure 7A,B) or IMQ (Figure 7A,B), consequently leading to an increase in the abundance of GSH in the skin tissue (Figure 7C). Moreover, si‐Slc31a1 treatment has been shown to rescue the reduced mRNA levels of Fdx1, Lias, Aco‐2, Sdhb, and Atp7a/b caused by MC903‐ and IMQ‐induced mouse models (Figure 7D). Additionally, si‐Slc31a1 administration led to a notable increase in the expression levels of the iron–sulfur cluster proteins LIAS and FDX1, while the oligomerization of the sulfur‐transfer protein DLAT was increased (Figure 7E). In addition, the application of si‐Slc31a1 effectively reversed the MC903‐ or IMQ‐mediated downregulation of Fth1 (Figure 7E), as well as reduced the increase in Fe^2+^ and lipid peroxidation levels in AD and psoriasis‐like (Figure 7F–G) mouse models. Taken together, these results suggest that inhibition of SLC31A1‐mediated cuproptosis alleviates inflammatory skin diseases.

## 3 Discussion

Cuproptosis has been documented to be strongly correlated with various inflammatory diseases, such as rheumatoid arthritis [32], osteoporosis [33], and Crohn's disease [34]. For example, cuproptosis‐related molecules, such as FDX1, LIAS, DLD, PDHA1, PDHB, DLAT, and LIPT1 were downregulated in lung tissue [35, 36]. Similarly, the expression of FDX1, DLAT, and PDHA1 was markedly decreased in dilated cardiomyopathy [37]. Moreover, cuproptosis has been implicated in the excessive survival and proliferation of immune cells, such as synovial fibroblast‐like cells, effector T cells, and macrophages, in rheumatoid arthritis [38]. Gene set enrichment analysis demonstrated a significant association between cuproptosis‐related gene FDX1 and immune‐related pathways [39]. In addition, cuproptosis has been found to play a significant role in modulating the cancer‐related microenvironment [40, 41], particularly in cancer immunotherapy [42, 43]. For instance, cuproptosis‐related genes are closely linked to immune cell infiltration in pancreatic cancer [44]. Additionally, DLAT has emerged as a potential immune biomarker for pancreatic adenocarcinoma [45]. Furthermore, several miRNAs and lncRNAs associated with cuproptosis genes have been found to have significant associations with the immune microenvironment [46, 47, 48].

In our study, we found that SLC31A1, a copper ion transporter, was significantly elevated in AD and psoriasis patient skin lesions, while inhibition of SLC31A1 expression attenuated MC903‐ or IMQ‐induced mouse dermatitis. SLC31A1 plays essential roles in various biological processes, with its primary functions associated with acetyl‐CoA metabolism, mitochondrial matrix functions, and acyltransferase enzyme activity. Moreover, the SLC31A1 expression was positively correlated with T helper 2 (Th2) cells and macrophages, but negatively correlated with plasmacytoid dendritic cells (pDCs), natural killer (NK) CD56^+^ bright cells, and CD8^+^ T cells [49]. In breast cancer, the expression of SLC31A1 shows a significant positive correlation with immune markers, notably STAT1 in Th1 cells and CCR8 in Treg cells. Furthermore, SLC31A1 positively regulates B cells, macrophages, and dendritic cells, thereby promoting tumor immune evasion [50].

We also found that FTH1 was downregulated in AD and psoriasis. FTH1, also known as ferritin heavy chain 1, is a protein involved in iron storage and homeostasis. A decrease in the levels of FTH1 can result in an imbalance in iron metabolism, leading to increased iron availability within cells. This excess iron can then contribute to the generation of ROS and lipid peroxidation, ultimately promoting ferroptosis [28, 51]. The occurrence of ferroptosis and the subsequent release of proinflammatory signals can trigger inflammatory processes. These processes can involve the activation of immune cells, release of cytokines and chemokines, and recruitment of additional immune cells to the affected site [29, 30, 52]. The overall consequence is an amplification of the inflammatory response. In addition, recent studies have shown that ferroptosis can promote a variety of skin inflammatory diseases, including psoriasis and AD [53, 54, 55, 56, 57]. Therefore, the decrease in FTH1 can potentially contribute to the increased level of ferroptosis, which in turn promotes skin inflammatory processes. Recent evidence has shown that excessive accumulation of copper ions could result in increasing iron toxicity and the development of oxidative stress [58]. Accumulating evidence has also shown that copper homeostasis influences ferroptosis in various diseases, including neurodegenerative diseases, immunological diseases, and cancer [59, 60, 61]. Copper has been shown to have unexpected effects in facilitating ferroptosis through the induction of giant autophagy/autophagic degradation via GPX4, whereas copper chelators blunted ferroptosis sensitivity but did not affect other types of cell death, such as apoptosis, necroptosis, and alkalosis [62]. Understanding this link between cuproptosis, ferroptosis, and inflammation may provide insights into the development of novel therapeutic strategies targeting these pathways to alleviate inflammatory conditions. Significantly our study indicated the inhibition of cuproptosis elevated FTH1 expression, but suppressed Fe^2+^ and lipid peroxidation levels, which suggested cuproptosis may affect the skin inflammation responses by regulating FTH1‐mediated ferroptosis. It was reported that the α‐KG affects the activity of demethylase, leading to the activation of lysine demethylase KDM5B and a decrease in H3K4me3 levels [31]. We found that SLC31A1‐mediated cuproptosis also regulated FTH1 by α‐KG/KDM5B/H3K4me3 axis to induce ferroptosis, which in turn leads to increased release of inflammatory factors, promotes Th17 differentiation, and ultimately accelerates the progression of skin inflammation. While our findings illuminate a novel pathogenic axis linking cuproptosis to ferroptosis in inflammatory skin diseases, several limitations merit consideration. First, the complex regulatory cascade upstream of SLC31A1 activation within the skin microenvironment remains incompletely defined. Future studies are needed to identify the specific triggers modulating SLC31A1 activity or expression to fully understand the initiation of this axis. Second, the skin comprises diverse cell types. In addition, while targeting SLC31A1/KDM5B/FTH1 is promising, translating this into therapies faces the challenge of potential systemic toxicity due to copper's essential roles. Developing localized delivery strategies or identifying downstream targets specific to the cuproptosis‐ferroptosis crosstalk could enhance therapeutic selectivity. Despite these limitations, our core findings regarding the central role of the α‐KG/SLC31A1/KDM5B/FTH1 axis in driving inflammation via cuproptosis‐ferroptosis signaling provide a strong foundation and valuable targets for future research and potential therapeutic development.

## 4 Conclusion

In summary, cuproptosis promotes ferroptosis by downregulating FTH1, which aggravates the symptoms of psoriasis and AD. Mechanically, cuproptosis promotes α‐KG accumulation, resulting in KDM5B‐mediated demethylation of H3K4me3, reducing FTH1 transcription level, facilitating ferroptosis, and leading to the release of inflammatory factors, thereby accelerating the progression of inflammatory skin diseases. Inhibition of cuproptosis could effectively alleviate psoriasis and AD‐like dermatitis, suggesting the novel therapeutic potential of targeting cuproptosis for clinical treatment.

## 5 Materials And Methods

### 5.1 Inflammatory Skin Disease Mouse Models and Treatment

Male BALB/c mice weighing 16–18 g and aged 6–8 weeks were housed in a specific pathogen‐free environment with a 12‐h light–dark cycle. The mice had ad libitum access to water and food. Animal care and experimental procedures were conducted in accordance with the ethical guidelines for experimental animal welfare and were approved by the ethical review board of the Experimental Animal Center at Central South University (Hunan, China) (202310040). To induce psoriasis‐like dermatitis, 0.1 mL of IMQ (Med‐shine

Pharmaceutical Co., Ltd.) was administered. For the construction of the AD‐like dermatitis model, 45 µmol/L of MC903 (Sigma) dissolved in ethanol was used. The experimental groups received the following interventions: TTM (Sigma) was administered via daily intraperitoneal injection (AD group: 20 mg/kg; psoriasis group: 40 mg/kg). Both groups additionally received daily topical application of si‐Slc31a1 (RiboBio) solution (2.5 nmol). The AD group exclusively received daily topical Fer‐1 (Targetmol) solution (0.8 mg/kg). Each group consisted of eight mice. Daily digital imaging was performed on the ears of the mice. After successful modeling, peripheral blood, skin lesions, and spleen samples were collected from the mice for subsequent experiments.

### 5.2 ScRNA Analysis of AD and Psoriasis

The scRNA‐seq datasets used in our study included GSE147424 (lesional/nonlesional skin biopsies taken from the extremities of five moderate‐to‐severe AD patients and seven matching controls) and GSE151177 (skin biopsy tissues from 13 psoriasis patients and five healthy volunteers) [21, 22]. Cells were filtered based on a gene expression count per cell between 100 and 5000 and a mitochondrial percentage per cell below 25. Read counts for each gene were normalized to 10,000 to generate transcripts per kilobase million (TPM)‐like values, and the two datasets were integrated by the Seurat package (version 4.1.1). After quality control and processing, we ran the analysis workflow by Seurat. First, we used “FindVariableFeatures” to obtain 2000 features by setting the parameter “selection.method” to “mvp” and “nfeatures” to 2000 and then scaled the data using the “ScaleData” function. Principal components analysis (PCA) was performed using the “RunPCA” function. The number of PCs was chosen by visualization plot with the “ElbowPlot” function. For the batch effect, we used the “RunHarmony” method to integrate the samples. Next, “RunUMAP(object, reduction = “harmony,” dims = 1:20)” was used, while clusters were calculated using the “FindClusters” function with a resolution of 0.4. The markers for identifying cells can be found in Table S1.

In this study, we defined a type of cell that exhibited an imbalance in copper homeostasis with SLC31A1 expression greater than 1 and ATP7B expression less than 1. Differentially expressed genes were identified with the “FindMarkers” function of the Seurat package, setting “logfc.threshold” to 0.25, “min.pct” to 0.1, and “test.use” to the Wilcox method. The cuproptosis‐related score was computed by the Seurat function “AddModuleScore.”

### 5.3 Transcriptome Data Analysis of AD and Psoriasis

For the RNA‐seq data analysis, we employed edgeR package for data preprocessing, while for the microarray data we followed standard chip processing pipelines to obtain expression profiles. After acquiring the expression matrices of cuproptosis‐related genes, we performed visualization using ggplot2 package. Specifically, we utilized boxplots to demonstrate the expression changes of these cuproptosis‐related genes across different disease states or treatment conditions.

### 5.4 Establishment of Clinical Prediction Models

Treatment response dataset information from psoriasis and AD patients was collected, and sequencing results of lesion site samples before treatment were screened (GSE69967 for psoriasis and GSE130588 for AD). Logistic regression was used to establish a univariate prediction model for SLC31A1 and FTH1 and a bivariate model for SLC31A1 + FTH1. The performance of the model was evaluated using ROC curves. The validation datasets were validated using medication data from other drugs using the ADA dataset (GSE85034).

### 5.5 Fe^2+^ Detection

To detect intracellular and mitochondrial Fe^2+^, FerroOrange and Mito‐FerroGreen (Dojindo) were used according to the manufacturer's protocol. The skin tissue was digested and ground to obtain a cell suspension. FerroOrange working solution, at a concentration of 1 µmol/L, was added to the cell suspension, followed by incubation at 37°C in a 5% CO_2_ incubator for 30 min. Flow cytometry was utilized to detect the cells, with the PE channel selected for analysis.

### 5.6 Lipid Peroxide Measurement

After the various treatments, cells were stained with 5 µmol/L Liperfluo (Dojindo) or 2 µmol/L C11‐BODIPY581/591 probe (Thermo Fisher Scientific) following the manufacturer's instructions. The mean fluorescence intensity of each group was normalized to that of the control group. Analysis of C11‐BODIPY581/591 fluorescence was performed using FACS analysis (BD Biosciences) and evaluated using FlowJo 7.6 software. Data were collected from a minimum of 30,000 cells.

### 5.7 Extraction of Human Primary KCs

We collected foreskin from 23 healthy individuals. Healthy adolescents aged 18–20 who underwent circumcision in the Department of Urology, Xiangya Hospital of Central South University were selected. Inclusion criteria were healthy individuals without any inflammatory skin diseases and reproductive system diseases. The subcutaneous tissue was removed and cut into tissue blocks of approximately 0.5 cm × 0.5 cm. Note that 10 mL of a separation solution containing 0.25% dispase II was added to the tissue and digested for 16 h until the epidermis and dermis of the skin tissue were separated.

Collecting the epidermis, it was rinsed three times with PBS and cut into 0.1 cm × 0.1 cm tissue blocks using sterile surgical scissors. Trypsin (Beyotime) was added to the tissue and digested at 37°C for 10 min. Digested tissue was removed from tissue blocks using a 70‐µm filter and centrifuged to collect precipitates. The precipitates were then cultured under conditions of 37°C, 5% CO_2_, and saturated humidity. This study was approved by the Medical Ethics Committee of Xiangya Hospital, Central South University (202308636), and all patients provided by the organization signed informed consent forms.

### 5.8 Cell Culture

The murine epidermal cell line JB6 and human KCs cell line HaCaT were purchased from American Type Culture Collection (ATCC). HaCaT and JB6 cells were cultured in Dulbecco's modified Eagle's medium (DMEM)/high glucose medium (Gibco) supplemented with 10% fetal bovine serum, and primary KCs were cultured in KC medium (Sciencell). Cells were cultured at a constant temperature of 37°C and 5% CO_2_.

### 5.9 ROS Level Detection

DCFH‐DA (Sigma) was added to the culture medium, with DMSO used as the solvent control. The cells were then incubated at 37°C for 45 min, followed by detection using flow cytometry.

### 5.10 Hematoxylin‐Eosin (H&E) and IHC Staining

Briefly, paraffin sections were processed by removing wax, stained with hematoxylin and eosin, and dehydrated to complete the H&E staining procedure (Sinopharm Chemical Reagent Co., Ltd.). IHC was performed with the following steps. The embedded skin tissue was initially deparaffinized and then subjected to antigen retrieval in citrate buffer using a microwave oven. The sections were incubated overnight at 4°C with the primary antibody and visualized the next day using DAB color rendering. This study was approved by the ethics committees of Xiangya Hospital of Central South University, Changsha, Hunan, China, and informed consent was obtained from all subjects (20180314 and 202212806).

### 5.11 Quantitative Real‐Time Polymerase Chain Reaction (qRT‒PCR)

RNAiso Plus reagent (Takara Bio Inc.) was utilized for total RNA extraction, while the PrimeScript RT reagent kit (Takara Bio Inc., Otsu, Shiga, Japan) was employed for cDNA synthesis. Two‐step real‐time RT‒PCR was performed using SYBR Green Reagent (Bimake). The results were analyzed using the 2^−ΔΔCt^ method, and the data are presented as the ratio relative to the control gene β‐actin.

### 5.12 Western Blotting

After extracting the protein from the cell lysate, the protein concentration was determined using the Bicinchoninic Acid Assay kit (Beyotime). Subsequently, the proteins were separated by electrophoresis on a 12% SDS‒PAGE gel, transferred onto a PVDF membrane, and blocked with 5% skim milk. The membrane was then incubated with the primary antibody overnight at 4°C, followed by incubation with the corresponding secondary antibody. Finally, the protein bands were visualized using an enhanced chemiluminescence (ECL) imaging system.

### 5.13 Multiparameter Flow Cytometry Experiments

Skin biopsy tissues were collected, and migrating cells were harvested. A portion of the harvested skin cells (5000–10,000 cells) was loaded onto the 10X Genomics single‐cell chip for analysis. Simultaneously, the remaining harvested skin cells were subjected to multiparameter flow cytometry using Th2 and Th17 cell panels. The harvested cells were washed and incubated on ice for 30 min with fluorochrome‐conjugated monoclonal antibodies specific to cell‐surface markers. The cells were then acquired using a BD LSR II flow cytometer (BD Biosciences) and analyzed using FlowJo software (TreeStar). The gating strategy is presented in Figure S2E.

### 5.14 Determination of Copper Ion Content

Skin tissue was ground and dissolved in PBS buffer solution. After treatment with ascorbic acid and 3,5‐dibromo‐PAESA, the absorbance of the resulting blue complex was measured at a wavelength of 600 nm to calculate the concentration of Cu^2+^.

### 5.15 Determination of Reduced GSH

The skin was accurately weighed, and normal saline solution was added, with a ratio of weight (g) to volume (mL) of 1:9, to prepare the tissue homogenate. After centrifugation, the supernatant was collected, and testing was performed according to the instructions provided (Nanjing Jiancheng Bioengineering Institute). The absorbance values of each well were measured at 405 nm using a microplate reader.

### 5.16 Metabolomics

The metabolites were extracted from cell residue with 1 mL precooled methanol/acetonitrile/water (v/v, 2:2:1) under sonication for 1 h in ice baths. The mixture was incubated at −20°C for 1 h followed by centrifugation at 16,000 *g*, 4°C for 20 min, and then transferred to the sampling vial for LC‐MS analysis. Additionally, to ensure data quality for metabolic profiling, quality control (QC) samples were prepared by pooling aliquots of all samples that were representative of the all samples under analysis, and used for data normalization. QC samples were prepared and analyzed with the same procedure as that for the experiment samples in each batch. Dried extracts were then dissolved in 50% acetonitrile. Each sample was filtered with a disposable 0.22 µm cellulose acetate and transferred into 2‐mL HPLC vials and stored at −80°C until analysis. The LC/MS portion of the platform was based on a Shimadzu Nexera X2 LC‐30AD system equipped with an Acquity UPLC HSS T3 column (1.8 µm 2.1 × 50 mm Column, Waters) and a triple quadruple mass spectrometer (5500 QTRAP, AB SCIEX). Metabolites were detected in electrospray negative‐ionization and positive‐ionization mode. The 5 µL samples were injected sequentially with LC autosampler. The Acquity UPLC HSS T3 column was heated to 40°C under a flow rate of 200 µL/min. A gradient was used to separate the compounds consisting of 0.1% formic acid aqueous solution (solvent A) and 100% acetonitrile (solvent B). The gradient started at 100% solvent A for 2.5 min and increased linearly to 70% solvent A over 9 min, and then increased linearly to 0% solvent A over 1 min followed by a 5.4 min hold before returning to the starting mixture during 0.1 min and re‐equilibrating for 2.5 min. QC samples were injected every several samples during acquisition. The MS conditions were set as follows: negative‐ionization: source temperature 550°C, ion source Gas1 (GAS1): 40, ion source Gas2 (GAS2): 50, curtain gas (CUR): 35, ion spray voltage floating (ISVF): 4500V; positive‐ionization: source temperature 550°C, ion source Gas1 (GAS1): 40, ion source Gas2 (GAS2): 50, curtain gas (CUR): 35, ion spray voltage floating (ISVF): 5500 V. Transition were detected by MRM mode. MultiQuant 3.0.2 software was used to extract the original MRM data of MT1000 KIT metabolites and obtain the peak area of each metabolite.

### 5.17 Chromatin Immunoprecipitation

Cross‐linked chromatin from human primary skin cells and imunoprecipitations were prepared as previously described, co‐incubated with H3K4me3 antibody and incubated overnight at 4°C. Note that 25 µL of Protein A Dynabeads were then added to each sample, and the mixtures were incubated at 4°C for 4 h. The beads were washed extensively and eluted with 1% SDS and 0.1 M NaHCO_3_. Cross‐linked samples were reversed by heating overnight at 65°C in the presence of 0.2 M NaCl. Samples were then treated with RNase A and proteinase K for 2 h, and DNA was recovered. Quantitative real‐time PCR corrected for primer efficiencies in the linear range was performed using SYBR Green I (Bimake). Primer 1, F: GTGCCCGTTTAGTGGAGTTG, R: AGAAACCGCACACGGAGCCA; Primer 2, F: TTTCCCTAGGGATGGGGACC, R: CTGCAGGTTTGTGAGCATCC; Primer 3, F: CTGGAAGCTAGACTTCCAAA, R: AGGCTGATCTCCAACTCCGA; Primer 4, F: GGCTAACTAACATGGCTAAA, R: TCTAGGATCAAGTTTAACCT.

### 5.18 Histone Extraction

Histone was extracted by histone isolation kit (Sigma). The cells were digested and counted, centrifuged at 200 *g* for 5 min, and the precipitation was collected. The precipitate was washed twice with PBS, centrifuged at 200 *g* for 5 min, and supernatant was removed. Note that 100 µL of cold extraction buffer was added to every 1 million cells for re‐suspension, and then the cells were incubated at 4°C for 2 h in an end‐to‐end rotator. After incubation, the specimen was centrifuged at 20,800 *g* for 10 min and the liquid supernatant was collected.

### 5.19 α‐KG Concentration Detection

The α‐KG concentration was detected using the α‐KG content detection kit (Solarbio). Extraction solution 1 was added according to the instructions, the homogenate was centrifuged in an ice bath for 10 min at 4°C at 12,000 rpm, 0.8 mL supernatant was taken, 0.15 mL extraction solution 2 was added slowly, slowly blowed and mixed until no bubbles are generated, centrifuged at 4°C at 12,000 rpm for 10 min, and supernatant to be measured was taken. The solarbio protocol for the next steps was followed.

### 5.20 Immunofluorescence Staining

Sections were deparaffinized and hydrated using xylene and a graded ethanol series. Antigen retrieval was performed with citrate buffer via heat treatment. Nonspecific binding sites were blocked with goat serum, and target primary antibody was incubated at 4°C overnight or at room temperature. Fluorescence‐conjugated secondary antibody (light‐protected) was applied at room temperature for 1 h. Nuclei was counterstained with DAPI. Slides were mounted with antifade mounting medium. Images were acquired immediately using fluorescence microscopy. The slides that were protected from light at 4°C or −20°C were stored.

### 5.21 Statistical Analyses

All data were analyzed using GraphPad Prism 8.0 and ImageJ 1.8.0 software. The measurement data obtained from three or more independent repeated experiments are expressed as the mean ± standard deviation (mean ± SD). Paired Student's *t* test or one‐way analysis of variance (ANOVA) was employed for data analysis, with *p* < 0.05 considered statistically significant. The significance levels were denoted as follows: *p < 0.05, **p < 0.01, ***p < 0.001, and ****p < 0.0001.

## Author Contributions

J.L., C.P., X.C., F.Y.L., and P.Y. designed the experiments. P.Y., K.X.L., and R.X.Y. conducted the experiments and analyzed the data. S.F.L. performed the bioinformatics analysis. X.Q.Y., C.F., S.H.M., and G.M.W. provided technical support. P.Y., K.X.L., F.Y.L., R.X.Y., and C.P. wrote the paper. All authors reviewed the manuscript.

## Ethics Statement

Animal care and experimental procedures were conducted in accordance with the ethical guidelines for experimental animal welfare and were approved by the ethical review board of the Experimental Animal Center at Central South University (Hunan, China) (approval number 202310040). This study was approved by the Medical Ethics Committee of Xiangya Hospital, Central South University (approval number 202308636), and all patients provided by the organization signed informed consent forms.

## Conflicts of Interest

The authors declare no conflicts of interest.

## Supporting information




**Figure S1**: scRNA analysis of AD & Psoriasis. (A) Data analysis process; (B) Clustering analysis in AD and psoriasis; (C) Cell type in AD and psoriasis; (D) Dot plot displaying expression levels of cluster‐defining genes; (E) Fraction of cell population of different patients; (F) Expression of Slc31a1 in mice detected by IFA. n = 3. Scale bar = 200 µm.
**Figure S2**: TTM alleviate AD and psoriasis‐like dermatitis. (A) Expression of Fdx1 in mice detected by IFA. n = 3. Scale bar = 200 µm; (B) Mice were treated with ethanol and MC903 for 12 days (discontinued on day 6 and day 7) to construct the AD‐like dermatitis model. Mice were treated with Vaseline and IMQ for 7 days to establish psoriasis‐like dermatitis. Mice were treated with TTM (20 mg/kg in AD, 40 mg/kg in psoriasis) by intragastric administration every day. Spleen index (Spleen weight/mouse weight) of each group; (C) Body weight of mice in each group. n = 5; (D) Mice were treated with TTM (0 mg/kg, 20 mg/kg, 40 mg/kg) by intragastric administration for 12 day. H&E staining of the heart, liver, spleen, lung and kidney in mice. Scale bars = 200 µm; (E) The gating strategy of flow cytometry.
**Figure S3**: Fer‐1 alleviate AD‐like dermatitis. (A) Mice were treated with ethanol and MC903 for 12 days (discontinued on day 6 and day 7) to construct the AD‐like dermatitis model. Mice were treated with Fer‐1 (0.8 mg/kg in AD) by intraperitoneal injection every day; (B) Body weight of mice in each group; (C) Mice were treated with ethanol and MC903 for 12 days (discontinued on day 6 and day 7) to construct the AD‐like dermatitis model. Mice were treated with Fer‐1 (0.8 mg/kg in AD) by intraperitoneal injection every day. Phenotypic presentation and H&E staining as well as statistical analysis of the epidermal thickness of the ears in mice. Scale bars = 200 µm; (D) The severity scoring of skin lesions, the ear thickness, dermatitis scores and erythema as well as the statistical analysis were used to evaluating AD; (E) mRNA levels of inflammatory factors (Il4, Il5, Tslp were used for evaluating AD) associated with dermatitis in each group by qRT‐PCR. n = 5. * *p*<0.05, *** *p*<0.001.
**Figure S4**: α‐KG inhibits FTH1 expression through demethylation of H3K4me3. (A) Classification of components for detecting metabolites; (B) According to the OPLS‐DA model variable importance for the projection, measure the intensity and explanatory power of the expression patterns of each metabolite on the classification discrimination of each group; (C) Abundance analysis of differential metabolites (α‐KG); (D) The pathway impact of involved in differential metabolites; (E) The content of α‐KG in KCs after treated with si‐mock, si‐SLC31A1 or IL17A for 48 h; (F) Mice were treated with Vaseline and IMQ for 7 days to establish psoriasis‐like dermatitis. GSK467 was injected into the abdomen every other day (10 mg/kg). Body weight of mice in each group; (G) The erythema, scales and infiltration were used to evaluating the severity of skin inflammatory; (H) Design the primers for ChIP experiment; n = 5, * *p*<0.05, ** *p*<0.01, *** *p*<0.001.
**Figure S5**: Knockdown of Slc31a1 alleviate psoriasis‐like dermatitis. (A) Expression of Slc31a1 and Fth1 after knocking down Slc31a1 in JB6 cells by qRT‐PCR. (B) Mice were treated with ethanol and MC903 for 12 days (discontinued on day 6 and day 7) to construct the AD‐like dermatitis model. Mice were treated with Vaseline and IMQ for 7 days to establish psoriasis‐like dermatitis. Si‐Slc31a1 was applied to the back skin (2.5 nmol). Spleen index (Spleen weight/mouse weight) in each group. (C) The erythema, scales, infiltration and PASI scores were used to evaluating the severity of skin inflammatory. n = 5. * *p*<0.05, ** *p*<0.01, *** *p*<0.001.
**Table S1**: Markers to identify cell types.

## Data Availability

Some or all data, models, or code generated or used during the study are available from the corresponding author by request.
