# Supplementary material for: Cuproptosis Facilitates Chronic Skin Inflammation by Regulating the α‐Ketoglutarate/H3K4me3/Ferritin Heavy Chain 1 Signaling Pathway‐Mediated Ferroptosis
Source: MedComm (2020). 2025 Oct 7;6(10):e70425. doi: 10.1002/mco2.70425 (PMC12501494; doi:10.1002/mco2.70425)
Supplement: Supplementary file 1 — Figure S1: scRNA analysis of AD & Psoriasis. (A) Data analysis process; (B) Clustering analysis in AD and psoriasis; (C) Cell type in AD and psoriasis; (D) Dot plot displaying expression levels of cluster‐defining genes; (E) Fraction of cell population of different patients; (F) Expression of Slc31a1 in mice detected by IFA. n = 3. Scale bar = 200 µm. Figure S2: TTM alleviate AD and psoriasis‐like dermatitis. (A) Expression of Fdx1 in mice detected by IFA. n = 3. Scale bar = 200 µm; (B) Mice were treated with ethanol and MC903 for 12 days (discontinued on day 6 and day 7) to construct the AD‐like dermatitis model. Mice were treated with Vaseline and IMQ for 7 days to establish psoriasis‐like dermatitis. Mice were treated with TTM (20 mg/kg in AD, 40 mg/kg in psoriasis) by intragastric administration every day. Spleen index (Spleen weight/mouse weight) of each group; (C) Body weight of mice in each group. n = 5; (D) Mice were treated with TTM (0 mg/kg, 20 mg/kg, 40 mg/kg) by intragastric administration for 12 day. H&E staining of the heart, liver, spleen, lung and kidney in mice. Scale bars = 200 µm; (E) The gating strategy of flow cytometry. Figure S3: Fer‐1 alleviate AD‐like dermatitis. (A) Mice were treated with ethanol and MC903 for 12 days (discontinued on day 6 and day 7) to construct the AD‐like dermatitis model. Mice were treated with Fer‐1 (0.8 mg/kg in AD) by intraperitoneal injection every day; (B) Body weight of mice in each group; (C) Mice were treated with ethanol and MC903 for 12 days (discontinued on day 6 and day 7) to construct the AD‐like dermatitis model. Mice were treated with Fer‐1 (0.8 mg/kg in AD) by intraperitoneal injection every day. Phenotypic presentation and H&E staining as well as statistical analysis of the epidermal thickness of the ears in mice. Scale bars = 200 µm; (D) The severity scoring of skin lesions, the ear thickness, dermatitis scores and erythema as well as the statistical analysis were used to evaluating AD; (E) mRNA level [file MCO2-6-e70425-s001.docx]

**Cuproptosis facilitates chronic skin inflammation by regulating the α-ketoglutarate/H3K4me3/Ferritin Heavy Chain 1 signaling pathway-mediated ferroptosis**

Pian Yu ^1,2,3,4,5 #^, Kaixuan Li ^1,2,3,4,5,6 #^, Shifu Luo ^7,8,9^ ^#^, Rongxuan Yan ^1,2,3,4,5#^, Xiaoqing Yi ^1,2,3,4,5^, Chi Fang ^1,2,3,4,5^, Sihui Ma ^1,2,3,4,5^, Guanming Wang ^1,2,3,4,5^, Fanyan Luo ^6^ *, Xiang Chen ^1,2,3,4,5^ *, Cong Peng ^1,2,3,4,5^ *, Jie Li ^1,2,3,4,5^ *

1. The Department of Dermatology, Xiangya Hospital, Central South University

2. Hunan Key Laboratory of Skin Cancer and Psoriasis, Hunan Engineering Research Center of Skin Health and Disease, Xiangya Hospital, Central South University

3. Furong Labratory, Changsha, Hunan, China

4. National Engineering Research Center of Personalized Diagnostic and Therapeutic Technology

5. National Clinical Research Center for Geriatric Disorders, Xiangya Hospital, Central South University

6. The Department of Cardiovascular Surgery, Xiangya Hospital, Central South University

7. Faculty of Health Sciences, University of Macau, Taipa, Macao SAR 999078, China

8. Shenzhen Institute of Advanced Technology, Chinese Academy of Sciences, Shenzhen, Guangdong, China

9. Faculty of Computer Science and Control Engineering, Shenzhen University of Advanced Technology, Shenzhen, 518107, Guangdong, China

**# These authors contributed equally to this work.**

***These authors contributed equally to this work.**

*** Corresponding author:**

Jie Li, Department of Dermatology, Xiangya Hospital, Central South University, Xiangya Road #87, Changsha, Hunan, China, 410008. E-mail: [xylijie@csu.edu.cn](mailto:xylijie@csu.edu.cn);

Cong Peng, Department of Dermatology, Xiangya Hospital, Central South University, Xiangya Road #87, Changsha, Hunan, China, 410008. E-mail: [pengcongxy@csu.edu.cn](mailto:pengcongxy@csu.edu.cn);

Xiang Chen, Department of Dermatology, Xiangya Hospital, Central South University, Xiangya Road #87, Changsha, Hunan, China, 410008. E-mail: [chenxiangck@126.com](mailto:chenxiangck@126.com);

Fanyan Luo, Department of Cardial Surgery, Xiangya Hospital, Central South University, Xiangya Road #87, Changsha, Hunan, China, 410008. E-mail: drlfy@csu.edu.cn.

**Supplementary figures & legends**


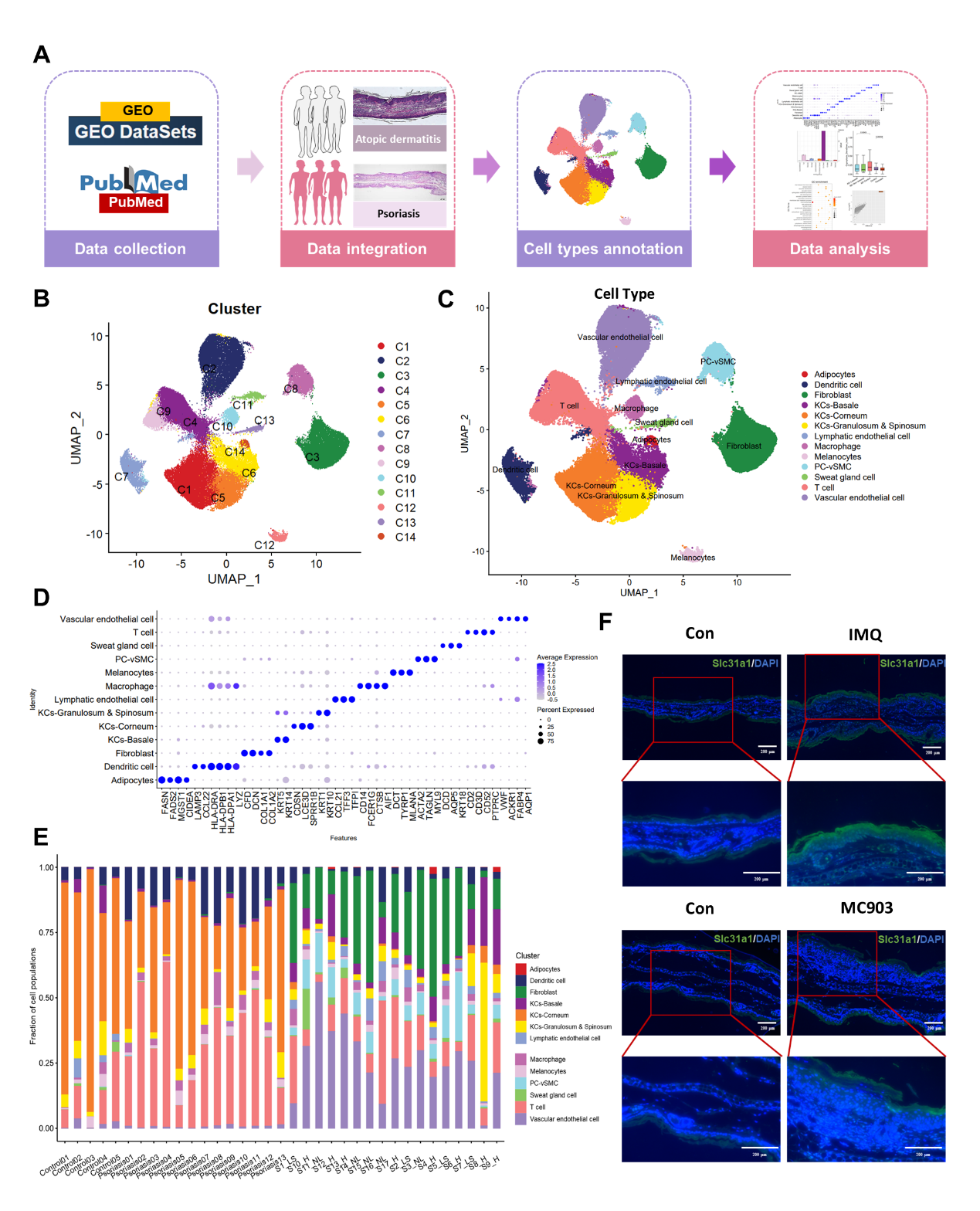


**Figure S1. scRNA analysis of AD & Psoriasis. (A)** Data analysis process; **(B)** Clustering analysis in AD and psoriasis; **(C)** Cell type in AD and psoriasis; **(D)** Dot plot displaying expression levels of cluster-defining genes; **(E)** Fraction of cell population of different patients; **(F)** Expression of Slc31a1 in mice detected by IFA. n = 3. Scale bar = 200μm.


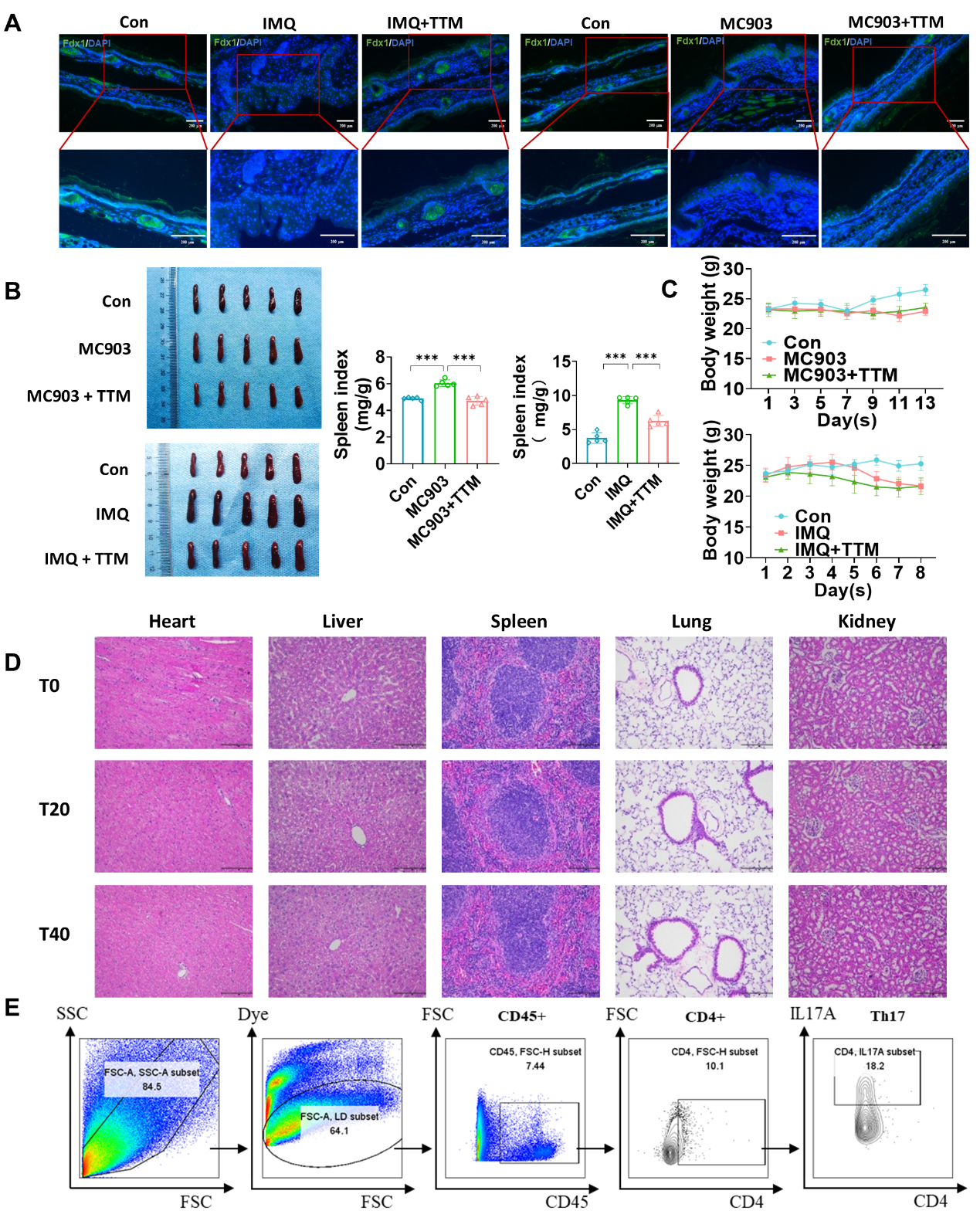


**Figure S2. TTM alleviate AD and psoriasis-like dermatitis. (A)** Expression of Fdx1 in mice detected by IFA. n = 3. Scale bar = 200μm; **(B)** Mice were treated with ethanol and MC903 for 12 days (discontinued on day 6 and day 7) to construct the AD-like dermatitis model. Mice were treated with Vaseline and IMQ for 7 days to establish psoriasis-like dermatitis. Mice were treated with TTM (20 mg/kg in AD, 40 mg/kg in psoriasis) by intragastric administration every day. Spleen index (Spleen weight/mouse weight) of each group; **(C)** Body weight of mice in each group. n = 5; **(D)** Mice were treated with TTM (0 mg/kg, 20 mg/kg, 40 mg/kg) by intragastric administration for 12 day. H&E staining of the heart, liver, spleen, lung and kidney in mice. Scale bars = 200 μm; **(E)** The gating strategy of flow cytometry.


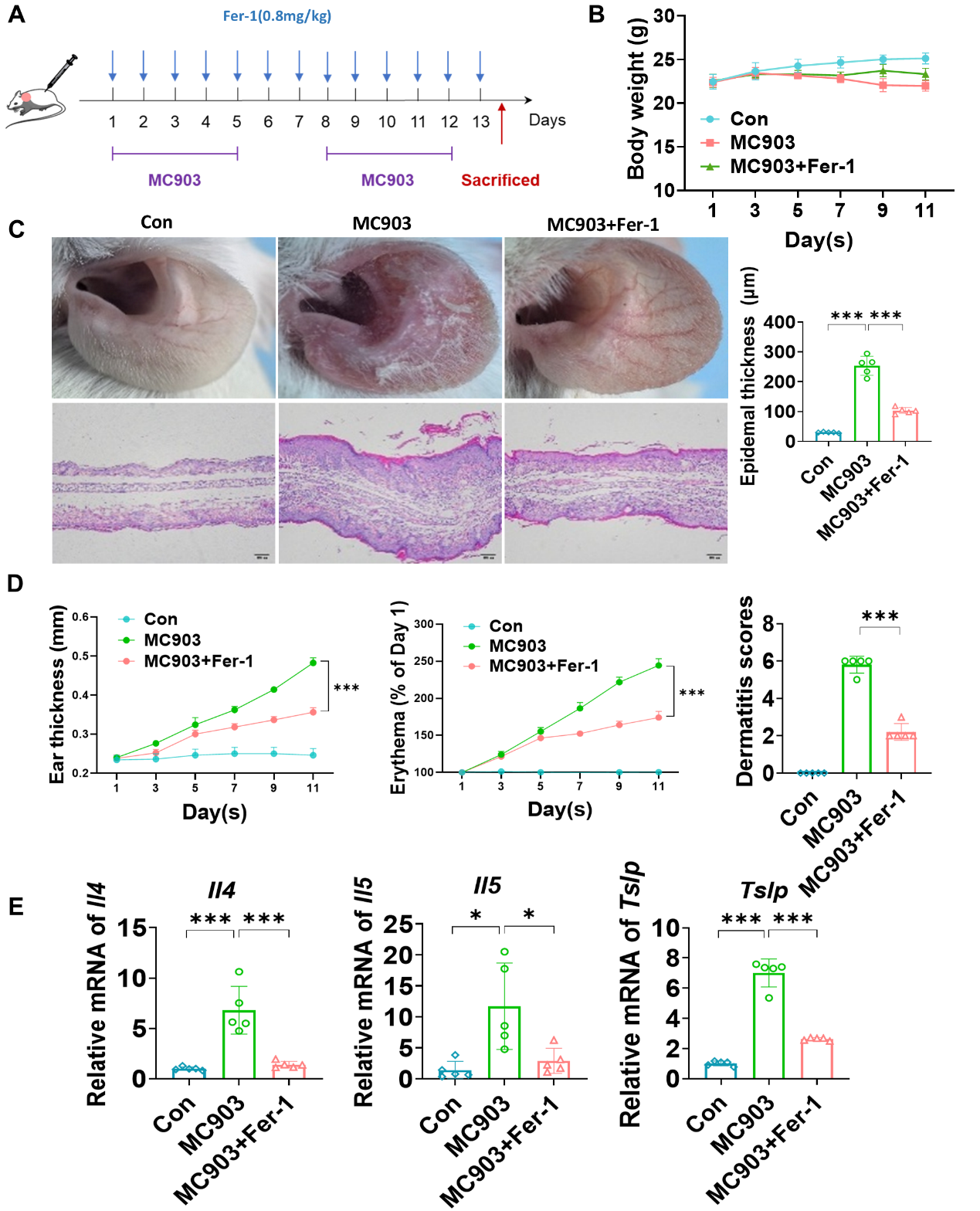


**Figure S3. Fer-1 alleviate AD-like dermatitis.** **(A)** Mice were treated with ethanol and MC903 for 12 days (discontinued on day 6 and day 7) to construct the AD-like dermatitis model. Mice were treated with Fer-1 (0.8 mg/kg in AD) by intraperitoneal injection every day; **(B)** Body weight of mice in each group; **(C)** Mice were treated with ethanol and MC903 for 12 days (discontinued on day 6 and day 7) to construct the AD-like dermatitis model. Mice were treated with Fer-1 (0.8 mg/kg in AD) by intraperitoneal injection every day. Phenotypic presentation and H&E staining as well as statistical analysis of the epidermal thickness of the ears in mice. Scale bars = 200 μm; **(D)** The severity scoring of skin lesions, the ear thickness, dermatitis scores and erythema as well as the statistical analysis were used to evaluating AD; **(E)** mRNA levels of inflammatory factors (Il4, Il5, Tslp were used for evaluating AD) associated with dermatitis in each group by qRT-PCR. n = 5. * *p*＜0.05, *** *p*＜0.001.


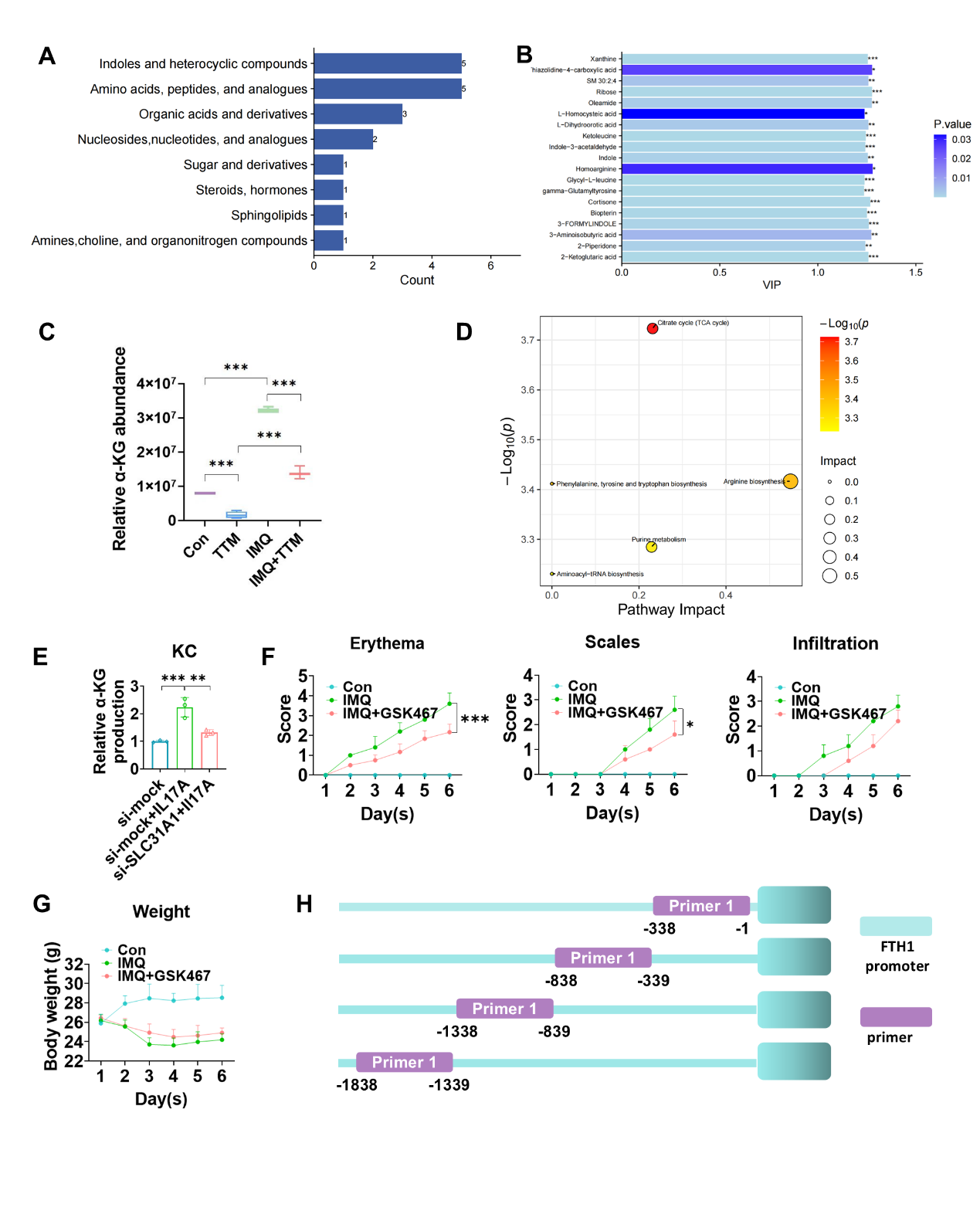


**Figure S4. α-KG inhibits FTH1 expression through demethylation of H3K4me3. (A)** Classification of components for detecting metabolites; **(B)** According to the OPLS-DA model variable importance for the projection, measure the intensity and explanatory power of the expression patterns of each metabolite on the classification discrimination of each group; **(C)** Abundance analysis of differential metabolites (α-KG); **(D)** The pathway impact of involved in differential metabolites; **(E)** The content of α-KG in KCs after treated with si-mock, si-SLC31A1 or IL17A for 48 h; **(F)** Mice were treated with Vaseline and IMQ for 7 days to establish psoriasis-like dermatitis. GSK467 was injected into the abdomen every other day (10 mg/kg). Body weight of mice in each group; (**G**) The erythema, scales and infiltration were used to evaluating the severity of skin inflammatory; **(H**) Design the primers for ChIP experiment; n = 5, * *p*＜0.05, ** *p*＜0.01, *** *p*＜0.001.


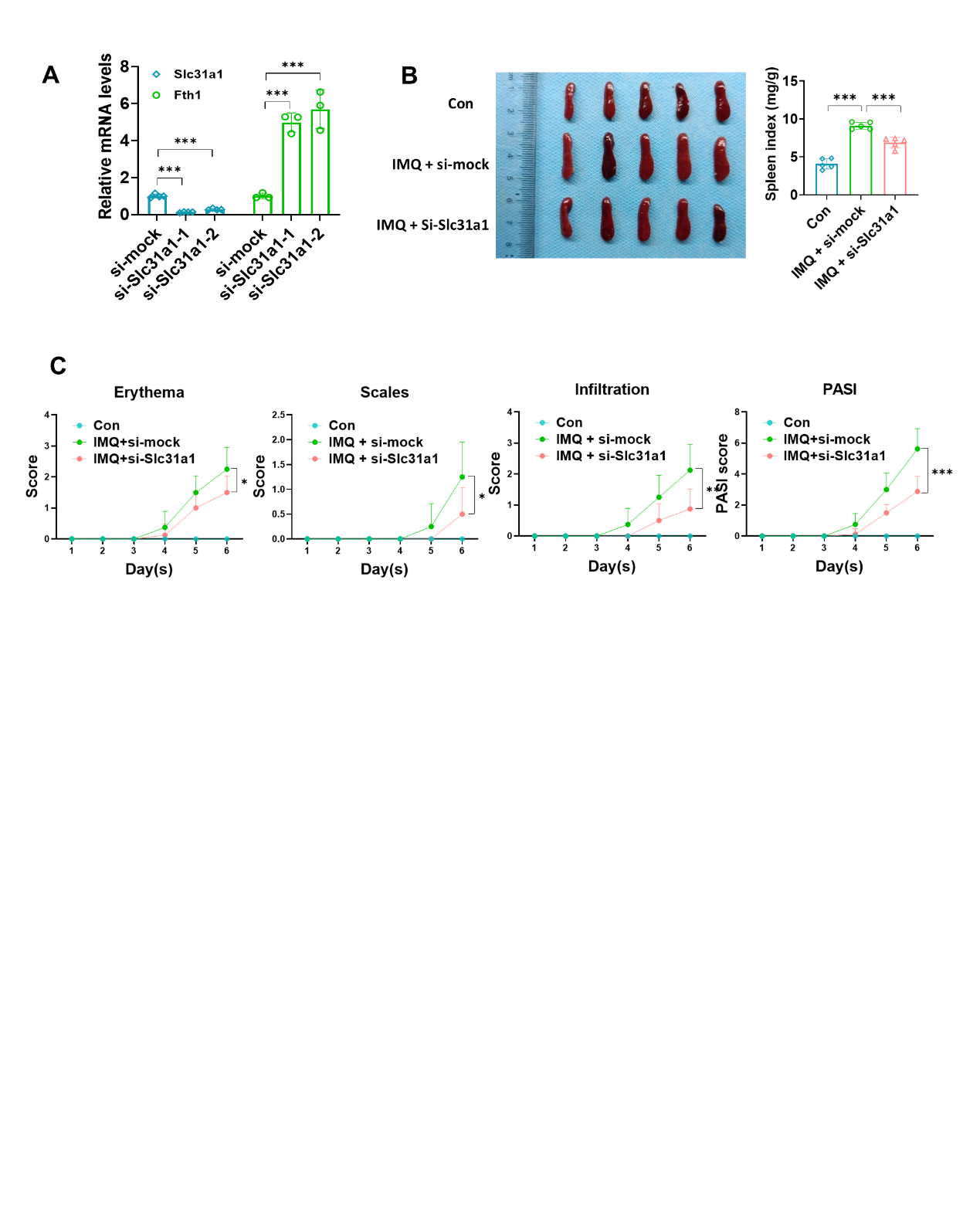


**Figure S5. Knockdown of Slc31a1 alleviate psoriasis-like dermatitis. (A)** Expression of Slc31a1 and Fth1 after knocking down Slc31a1 in JB6 cells by qRT-PCR. **(B)** Mice were treated with ethanol and MC903 for 12 days (discontinued on day 6 and day 7) to construct the AD-like dermatitis model. Mice were treated with Vaseline and IMQ for 7 days to establish psoriasis-like dermatitis. Si-Slc31a1 was applied to the back skin (2.5 nmol). Spleen index (Spleen weight/mouse weight) in each group. **(C)** The erythema, scales, infiltration and PASI scores were used to evaluating the severity of skin inflammatory. n = 5. * *p*＜0.05, ** *p*＜0.01, *** *p*＜0.001.

**Table S1**. **Markers to identify cell types**

| Cluster | CellType | Markers |
| --- | --- | --- |
| C1 | KCs-Corneum | CDSN, LCE3D, SPRR1B, CRCT1, CNFN |
| C2 | Vascular endothelial cell | VWF, ACKR1, FABP4, AQP1 |
| C3 | Fibroblast | CFD, DCN, COL1A1, COL1A2 |
| C4 | T cell | CD3D, CD52, TRAC, TRBC1, PTPRC |
| C5 | KCs-Granulosum & Spinosum | KRT1, KRT10 |
| C6 | KCs-Basale | KRT5, KRT14 |
| C7 | Dendritic cell | LAMP3, CCL22, HLA-DRA, HLA-DPB1, HLA-DPA1, LYZ |
| C8 | PC-vSMC | ACTA2, TAGLN, MYL9 |
| C9 | T cell | CD3D, CD52, PTPRC |
| C10 | Macrophage | CD14, AIF1, FCER1G, CTSB |
| C11 | Lymphatic endothelial cell | CCL21, TFF3, LYVE1 |
| C12 | Melanocytes | DCT, TYRP1, MLANA |
| C13 | Sweat gland cell | DCD, AQP5 |
| C14 | Adipocytes | FASN, FADS2, MGST1, CIDEA |
